# Functional diversification despite structural congruence in the HipBST toxin-antitoxin system of *Legionella pneumophila*

**DOI:** 10.1128/mbio.01510-23

**Published:** 2023-10-11

**Authors:** Jordan D. Lin, Peter J. Stogios, Kento T. Abe, Avril Wang, John MacPherson, Tatiana Skarina, Anne-Claude Gingras, Alexei Savchenko, Alexander W. Ensminger

**Affiliations:** 1 Department of Molecular Genetics, University of Toronto, Toronto, Ontario, Canada; 2 Department of Chemical Engineering and Applied Chemistry, University of Toronto, Toronto, Ontario, Canada; 3 Lunenfeld-Tanenbaum Research Institute, Sinai Health, Toronto, Ontario, Canada; 4 Department of Microbiology, Immunology and Infectious Diseases, University of Calgary, Calgary, Alberta, Canada; 5 Center for Structural Genomics of Infectious Diseases (CSGID), University of Calgary, Calgary, Alberta, Canada; 6 Department of Biochemistry, University of Toronto, Toronto, Ontario, Canada; University of Michigan-Ann Arbor, Ann Arbor, Michigan, USA

**Keywords:** *Legionella pneumophila*, toxin-antitoxin system, HipBA, HipBST, split hipA, kinase

## Abstract

Toxin-antitoxin (TA) systems are abundant genetic modules in bacterial chromosomes and on mobile elements. They are often patchily distributed, as a consequence of horizontal exchange, and the breadth of their functionality in bacterial physiology remains unknown. Here, we characterize a TA system in *Legionella pneumophila* that is highly conserved across *Legionella* species. This system is distantly related to *Escherichia coli* HipBST, and we demonstrate that it is a functional tripartite TA system (denoted HipBST_Lp_). We identify HipBST_Lp_ homologs in diverse taxa, yet in the Gammaproteobacteria, these are almost exclusively found in *Legionella* species. Notably, the toxin HipT_Lp_ was previously reported to be a pathogenic effector protein that is translocated by *L. pneumophila* into its eukaryotic hosts. Contrary to this, we find no signal of HipT_Lp_ translocation beyond untranslocated control levels and make several observations consistent with a canonical role as a bacterial toxin. We present structural and biochemical insights into the regulation and neutralization of HipBST_Lp_, and identify key variations between this system and HipBST_Ec_. Finally, we show that the target of HipT_Lp_ is likely not conserved with any characterized HipA or HipT toxin. This work serves as a useful comparison of a TA system across bacterial species and illustrates the molecular diversity that exists within a single TA family.

**IMPORTANCE:**

Toxin-antitoxin (TA) systems are parasitic genetic elements found in almost all bacterial genomes. They are exchanged horizontally between cells and are typically poorly conserved across closely related strains and species. Here, we report the characterization of a tripartite TA system in the bacterial pathogen *Legionella pneumophila* that is highly conserved across *Legionella* species genomes. This system (denoted HipBST_Lp_) is a distant homolog of the recently discovered split-HipA system in *Escherichia coli* (HipBST_Ec_). We present bioinformatic, molecular, and structural analyses of the divergence between these two systems and the functionality of this newly described TA system family. Furthermore, we provide evidence to refute previous claims that the toxin in this system (HipT_Lp_) possesses bifunctionality as an *L. pneumophila* virulence protein. Overall, this work expands our understanding of the split-HipA system architecture and illustrates the potential for undiscovered biology in these abundant genetic elements.

## INTRODUCTION

Bacterial genomes are a mosaic of genes that are highly conserved and often critical for growth (the core genome) or variable and typically non-essential (the accessory genome). The accessory genome is composed of genes frequently acquired through horizontal transfer, and the plasticity of this portion of the genome can promote organismal diversification while maintaining robustness to changing selective pressures (1). The contribution of accessory genomic elements to bacterial physiology is, therefore, of critical importance to understand, in particular with regard to genes that are considered accessory but demonstrate patterns of significant conservation across species (2).

Toxin-antitoxin (TA) systems are abundant and widely distributed components of the accessory genome (3). They are typically bipartite genetic modules found in nearly all bacterial chromosomes—often in multiple types and copies—as well as on mobile DNA elements (4, 5). Despite their widespread occurrence, these genetic systems are typically poorly conserved across related strains and species (5, 6). Several functions have been described for TAs, with substantial evidence for their role in phage defense and DNA stabilization (7), while considerable controversy surrounds their contribution to bacterial persistence (3, 8). TA systems are broadly divided into eight major groups based on antitoxin activity and molecule type (7, 9), with the most heavily studied being the type II systems. Type II TA systems are composed of a protein toxin that can reversibly arrest cellular growth and a protein antitoxin that neutralizes the toxin through direct physical interaction. The toxin is typically refractory to proteolytic degradation, whereas the antitoxin is often rapidly degraded by the cell and needs to be frequently replenished (10). This leads to cellular addiction to the TA system—a property that allows them to act as selfish elements and consequently, to stabilize mobile DNA (11).

Numerous mechanisms of cell toxification and toxin neutralization have been reported for type II systems (12). Genetic diversification has also been observed within the same TA system, such as the recent discovery of a split-kinase variant of the well-studied HipBA system in *Escherichia coli* (13). HipBA is composed of the HipA kinase toxin and HipB antitoxin and has been implicated in regulating bacterial persistence (14, 15). The newly described TA family HipBST is related to HipBA but displays a tripartite architecture: an N-terminal subdomain in the HipA kinase is encoded by a separate protein (HipS) and this functions as the antitoxin in the system (13). The catalytic core of HipA is expressed as a single protein (HipT) that retains toxin activity, while the canonical antitoxin HipB appears to transcriptionally regulate the locus. This unusual tripartite variant is, therefore, considerably divergent from the HipBA system despite their relatedness.

Most knowledge of TA biology has been gleaned from studying a small number of bacterial species, but given their modularity and portability, it remains unclear whether homologous systems function similarly within different bacterial hosts (7). To address this, we sought to investigate the TA landscape within the intracellular pathogen *Legionella pneumophila. L. pneumophila* is a Gram-negative environmental bacterium found within most global freshwater environments, where it parasitizes numerous protozoan species (16). To maintain this host range, *L. pneumophila* utilizes an arsenal of translocated virulence proteins (termed “effectors”) to transform the host cell into a replicative niche (17). The genome of *L. pneumophila* contains the largest known assemblage of effectors (~10% of genes), many of which contain homology to eukaryotic proteins and have likely been acquired via horizontal gene transfer from its hosts (18). Conversely, it encodes only a small number of predicted TA systems (19) and is largely devoid of mobile elements (such as prophage), which contribute to TA dissemination (7). We, therefore, wondered whether unique TA biology would be found in a species with the genomic constraints of an intracellular pathogen that has prioritized the acquisition of foreign genetic material from its hosts.

Here, we report the characterization of a TA system in *L. pneumophila* that is highly conserved across *Legionella* species genomes. This system is distantly related to the HipBST module in *E. coli* (herein referred to as HipBST_Lp_ and HipBST_Ec_, respectively) and the toxin HipT_Lp_ was previously reported to be an effector that is translocated into the eukaryotic host (20). Recently, a bi-functional role for HipT_Lp_ was proposed with activity in both the host and the bacterial cell (21). Contrary to this, we demonstrate that HipT_Lp_ is not translocated at a level greater than a negative (non-effector) control and has no obvious role within the eukaryotic cell. Instead, we show that *hipBST_Lp_* encodes a functional tripartite TA system with clear effects on bacterial growth. Interestingly, HipBST_Lp_ represents a heretofore undiscovered subclade of HipBST systems that are widely distributed outside of the Gammaproteobacteria class but are almost exclusively found in the Legionellales order within that group. We demonstrate that the toxin HipT_Lp_ is a kinase and report a survey of its phosphoproteome within intoxicated cells. We find that despite their shared architecture, HipT_Lp_ does not appear to target the same cellular substrate as HipT_Ec_ or any characterized HipA toxin. We additionally present structural and biochemical insights into the HipBST_Lp_ system, including its mechanism of neutralization and sites of divergence from HipBST_Ec_. Overall, this work provides a comparison of a TA system across distant bacterial species and emphasizes that generalizations about system functionality should be made with caution. Instead, there appears to be considerable evolutionary diversity that remains underexplored within these abundant genetic elements.

## RESULTS

### *Legionella pneumophila* encodes a highly conserved HipBST locus

**Fig 1:**
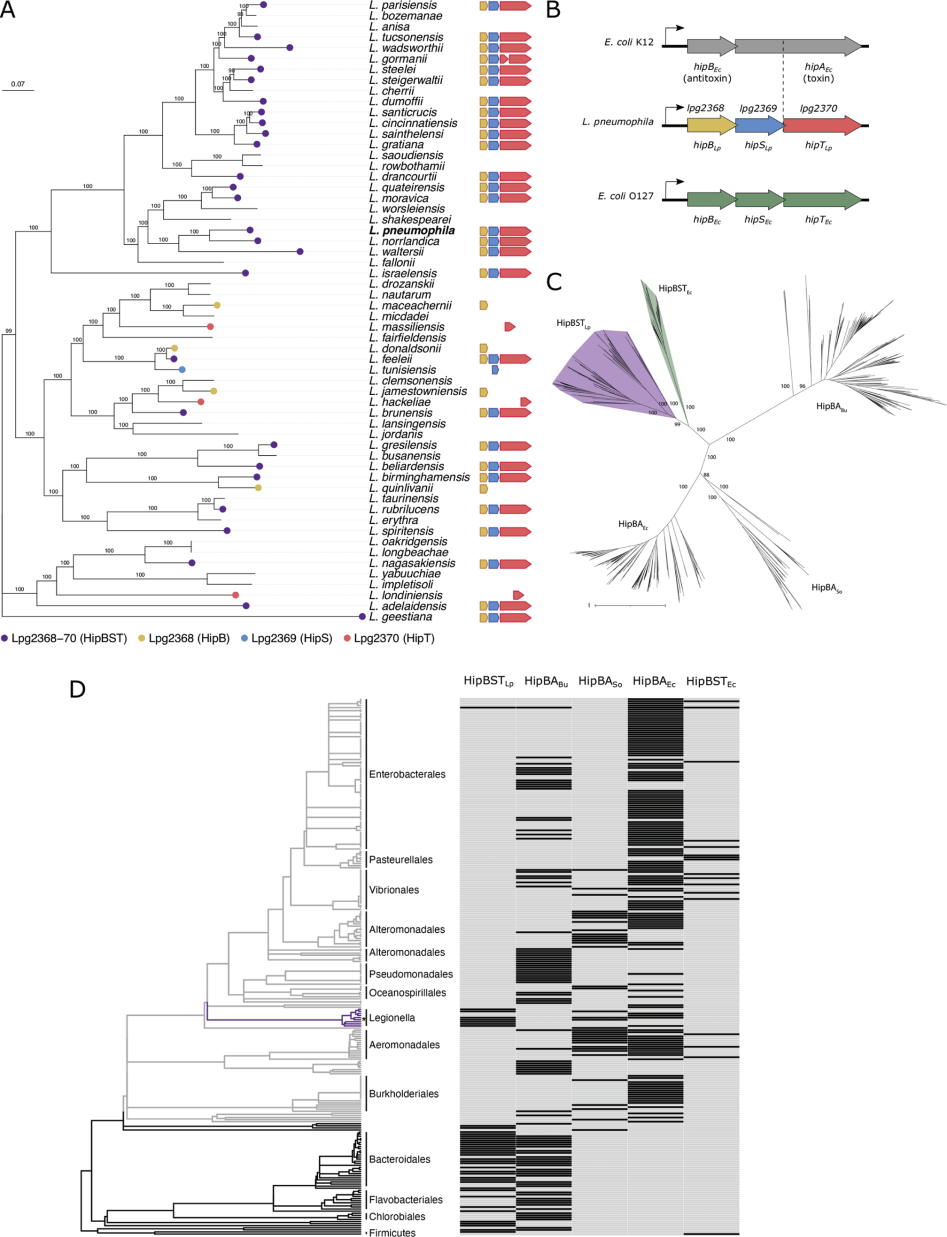
*Legionella* species contain a highly conserved and taxonomically restricted HipBST TA system. (**A**) A core genome phylogeny of 58 representative species in the *Legionella* genus (Table S4) showing the conservation of Lpg2368-70 (HipBST_Lp_) homologs. The *Legionella* core genome was determined using OrthoMCL (25) to identify ORFs with conservation across all 58 species. Amino acid sequences were aligned with MUSCLE (26), a phylogenetic tree was constructed with RAxML (27), and the tree was rooted with *Legionella geestiana* as the outgroup based on previous phylogenies (22, 23). Tree annotation and visualization were performed with ggtree (28) and FlaGs (29). The scale bar denotes substitutions per site. Bootstrap values are indicated for each node. (**B**) Schematic of the HipBA_Ec_, HipBST_Lp_, and HipBST_Ec_ TA systems. Lpg2368 has homology to the canonical antitoxin HipB, whereas Lpg2369 and Lpg2370 align to the N and C termini of the HipA toxin, respectively. (**C**) An unrooted phylogeny of HipBA and HipBST homologs retrieved from the NCBI Refseq database. Homology searching was performed using cblaster (30) with seed sequences from five different TA systems: *E. coli* K12 HipBA (HipBA_Ec_; NP_416025.1, NP_416024.1), *Shewanella oneidensis* MR-1 HipBA (HipBA_So_; AAN53783.2, AAN53784.1), *Bacteroides uniformis* HipBA (HipBA_Bu_; WP_149924066.1, WP_149924064.1), *E. coli* O127 HipBST (HipBST_Ec_; WP_000563102.1, WP_001346664.1, WP_001262465.1), and *L. pneumophila* HipBST (HipBST_Lp_; AAU28429, AAU28430, AAU28431). Retrieved sequences were clustered using MMseqs (31) to identify sequence-level representatives, aligned with MAFFT (32), and trimmed with trimAI (33). HipS/HipT sequences were concatenated prior to alignment with the HipA sequences, whereas HipB sequences were aligned separately. The phylogeny was constructed using IQ-TREE (34) and visualized with iTOL (35). The scale bar denotes substitutions per site. Bootstrap values are indicated for major nodes. Raw and sequence-level representative hits are provided in Table S5. (**D**) Distribution of HipBA and HipBST TA systems across diverse bacterial taxa. A bacterial phylogeny was retrieved from TimeTree (36) for species containing at least one system in our homology search (Table S6). These were filtered to remove species without a complete genome, to ensure that genome incompleteness did not influence system detection. The presence of a homolog for each system is indicated for each species and systems are ordered by similarity of taxonomic distribution. The Pseudomonadota phylum is colored light gray, the *Legionella* genus is colored purple, and *L. pneumophila* is indicated with an asterisk.

The genome of *L. pneumophila* has seven predicted TA loci in the Toxin-Antitoxin Database (19)—a comparatively small number relative to other studied bacterial species. We first investigated the distribution of these systems across 58 representative species of *Legionella* in the NCBI Refseq database (Table S4) to determine whether any showed a signature of high conservation. Surprisingly, despite the mobile nature of TA systems and their typically patchy distribution, we identified one system (*lpg2368-70*) that was present in nearly half of the *Legionella* species genomes (28/58 species; Fig. 1A). Additionally, eight other species had homologs of individual genes from this locus (Fig. 1A). This level of conservation is unusual for TA systems and was striking given the genomic diversity within this genus (22). Even effectors, which constitute a large portion of the *Legionella* accessory genome and are critical to pathogenicity, are poorly conserved across species (22, 23). Interestingly, *lpg2368-70* is similar to the well-characterized HipBA module (24) but possesses an atypical tripartite architecture in which the HipA homolog is split into two separate open reading frames (ORFs) (Fig. 1B). Given its substantial conservation across *Legionella* species (>80% sequence similarity across homologs) and unusual genetic organization, we chose to investigate this putative TA system and its functionality in *L. pneumophila*.

The *lpg2368-70* locus encodes three genes within a predicted operon (Fig. 1B) (37), with each ORF overlapping by 4 bp. The latter two genes were originally annotated as having homology to domains of the HipA_Ec_ toxin; the proteins encoded by *lpg2369* and *lpg2370* align with the N-terminus and C-terminus of HipA_Ec_, respectively. The third protein encoded by *lpg2368* has sequence similarity (~30%) to the antitoxin HipB_Ec_. These observations led us to hypothesize that Lpg2368-70 constituted a split-kinase TA system that was distantly related to HipBA. During the course of our studies, this was confirmed by the discovery of a similar system in *E. coli* (denoted HipBST_Ec_) (13). From this point forward, we refer to Lpg2368-70 as HipBST_Lp_. Despite this similar architecture, HipT_Lp_ bears only remote sequence similarity to either HipA_Ec_ (~28% identity) or the newly characterized HipT_Ec_ (~29% identity) (Fig. S1A), and HipB_Lp_ has no similarity to HipB_Ec_ of HipBST_Ec_. This may explain why the HipBST systems in *Legionella* species were not detected in previous work (24). While they exhibit broad differences at the sequence level, the core catalytic residues in HipA_Ec_ and HipT_Ec_ are conserved in HipT_Lp_ (Fig. S1A). HipBST_Lp_, therefore, provided the opportunity to compare two variants of the newly described HipBST family.

### The HipBST_Lp_ system is phylogenetically and taxonomically distinct from HipBST_Ec_

As HipBST_Lp_ was not identified previously, we sought to determine whether closely related modules were also undiscovered in other bacterial genomes and how they fit into the broader Hip phylogeny. We queried the NCBI Refseq database for HipBA and HipBST homologs using five diverse systems as seed sequences: HipBA from *E. coli* K-12 (HipBA_Ec_), HipBA from *Shewanella oneidensis* (HipBA_So_), HipBA from *Bacteroides uniformis* (HipBA_Bu_), HipBST from *E. coli* O127 (HipBST_Ec_), and HipBST from *L. pneumophila* (HipBST_Lp_). While HipBA_Ec_ and HipBA_So_ have been characterized previously (24, 38), HipBA_Bu_ was included in order to expand our search space, as this system was found in close proximity to a HipBST_Lp_ homolog and was itself quite divergent from the other HipA/HipT sequences (Fig. S1B). From this search, we identified 164 HipBA_Ec_, 37 HipBA_So_, 184 HipBA_Bu_, 31 HipBST_Ec_, and 86 HipBST_Lp_ sequence-level representative systems (Table S5) and used these sequences to construct a HipBST/HipBA phylogeny. Despite their shared tripartite architecture, HipBST_Lp_ homologs cluster in a separate subclade from the HipBST_Ec_ sequences (Fig. 1C), indicating system-level divergence even within the HipBST family. We discovered numerous HipBST_Lp_ homologs that are only distantly related to the 48 previously reported systems (24), thereby expanding the sequence space of HipBST. In fact, the majority (60/86) of our HipT_Lp_ representative sequences share less than 60% identity with any of the 48 sequences previously identified. In order to investigate the contribution of the individual protein components to the concatenated system phylogeny, we further constructed individual phylogenies for the HipB and HipST/HipA homologs separately (Fig. S1C). For this purpose and owing to the size disparity between HipS and HipA sequences, in addition to the likely constrained coevolution of HipS-HipT pairs, we treated HipST as a single concatenated sequence. Interestingly, while both component phylogenies largely agree with the system phylogeny, some incongruence is observed in the HipB phylogeny with regard to the clustering of HipB_So_-like sequences in the HipBST clade.

We next examined whether the various HipBA/HipBST homologs displayed any pattern of taxonomic distribution. To this end, we mapped the presence or absence of each system type onto a phylogeny of species that contained at least one system from our search and had a complete genome assembly in the Refseq database (Table S6). From this, we observed that HipBST_Lp_ homologs in the Gammaproteobacteria are almost exclusively found within the Legionellales order, whereas most detected tripartite systems in this class are instead homologs of HipBST_Ec_ (Fig. 1D; Fig. S1D). In contrast to their restricted distribution within the Gammaproteobacteria, HipBST_Lp_ homologs are widely distributed within distant taxonomic groups, particularly the FCB (Fibrobacterota, Chlorobiota, and Bacteroidota) superphylum. Interestingly, while HipBA_Bu_ homologs are prevalent throughout the bacterial phylogeny, homologs of HipBA_Ec_ and HipBA_So_ appear to be mostly restricted to the Pseudomonadota phylum. In summary, we used the previously undetected HipBST_Lp_ system to broaden the sequence space of known HipBST homologs. We observed that HipBST_Lp_ and HipBST_Ec_ sequences constitute distinct HipBST subclades, revealing considerably more genetic and taxonomic diversity within the HipBST TA family.

### Several lines of evidence highlight the pertinence of investigating the effects of HipT_Lp_ on the bacterial cell

**Fig 2:**
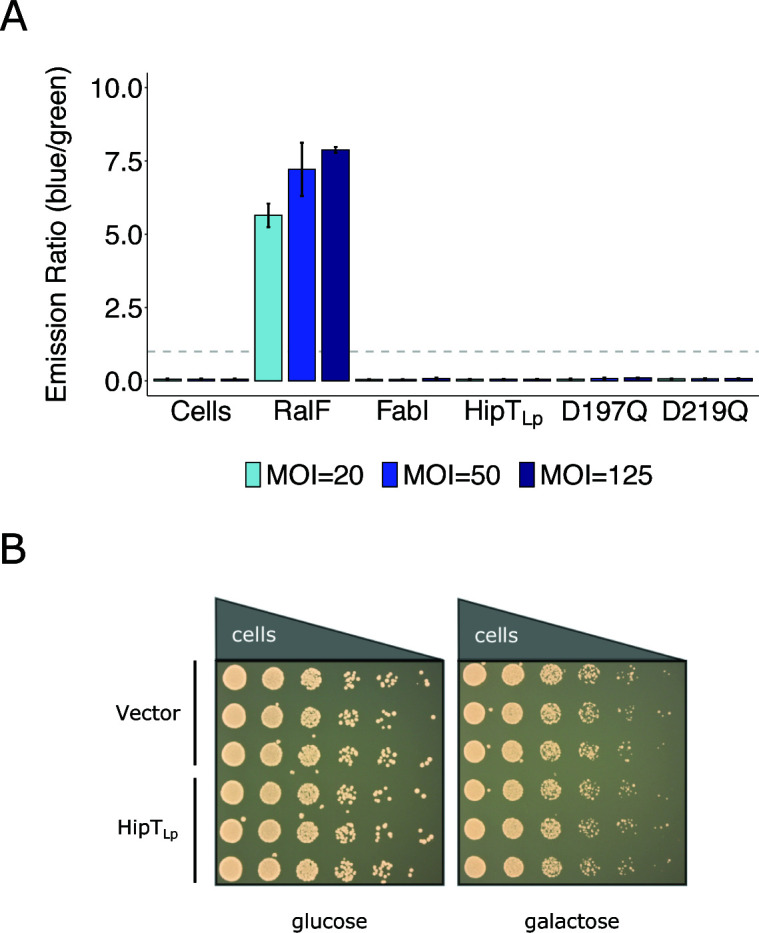
HipT_Lp_ translocation is not detectable above the levels of established negative controls. (**A**) TEM-1 β-lactamase translocation assays were performed with Lp02 cells infecting monolayers of differentiated U937 cells. Uninfected cells were used to determine background fluorescence and the cytosolic protein FabI was used as a negative control. The positive control used was the *L. pneumophila* effector RalF. Both controls, in addition to wild-type and mutant HipT_Lp_(D197Q, D219Q), were expressed from the pXDC61 vector (induced with 500 µM IPTG) as a fusion with the TEM-1 protein and infections were performed with the indicated MOIs. Quantified fluorescence is reported as the ratio of blue fluorescence (translocation) to green fluorescence (no translocation). The bar chart shows mean ± standard deviation of three biological replicates and is representative of three independent experiments. The dashed gray line indicates a blue/green fluorescence ratio of 1, which was used as a threshold for translocation. (**B**) Expression of HipT_Lp_ in *S. cerevisia*e BY4742 cells. Yeast cultures carrying HipT_Lp_ cloned into the pAG425GAL expression vector, or a vector-only control, were grown overnight and spotted in serial dilutions onto media containing either 2% glucose (repression) or 2% galactose (expression) and grown for 2 days. Spotting assays were performed in biological triplicate and images are representative of three independent experiments.

It has been previously reported that HipT_Lp_ is translocated by *L. pneumophila* into its eukaryotic host via its Type IV secretion system (20). This observation has significant potential to direct the future investigation of HipT_Lp_ towards activities against the host and away from any role as a canonical bacterial toxin. As such, we began our own functional analysis by carefully quantifying the levels of HipT_Lp_ translocation into host cells. Notably, translocation of HipT_Lp_ was previously detected at very low efficiency using the TEM-1 assay (21), in which TEM-1 β-lactamase is fused to a protein of interest and expressed in *L. pneumophila* cells infecting monolayers of differentiated U937 cells (39). Translocation is determined by monitoring emission from a fluorescent substrate within the host cells, which shifts from emitting green fluorescence to blue fluorescence upon cleavage of an internal β-lactam ring by the TEM-1 fusion. Given the low level of translocation previously reported, we sought to confirm this observation. Using identical methodology, we observed that the translocation signal of HipT_Lp_ is equivalent to that of the negative control FabI—a cytosolic protein with no function in the host (Fig. 2A). We observed the same negative results at several different multiplicities of infection (MOIs) and confirmed fusion protein expression by immunoblotting (Fig. S2). To test whether the toxicity of HipT_Lp_ was impacting its observed level of translocation, we additionally tested two substitutions in predicted catalytic residues (D197Q, D219Q) that are conserved with HipA_Ec_ (Fig. S1A) (40). However, no translocation signal above control levels was detected for these mutants (Fig. 2A) despite their increased expression levels (Fig. S2). Further arguing against its role as an effector, we observe that overexpression of HipT_Lp_ does not display any growth inhibitory effects in yeast (Fig. 2B), which is a common phenotype of effector expression (41). This is consistent with our identification of HipBST_Lp_ homologs within non-pathogenic bacterial species (Fig. 1D; Fig. S1D) and argues that, while effects against the eukaryotic host remain a tantalizing possibility, it remains pertinent to thoroughly investigate HipT_Lp_ for canonical toxin activity within the bacterial cell.

### HipBST_Lp_ is a functional tripartite toxin-antitoxin system

**Fig 3:**
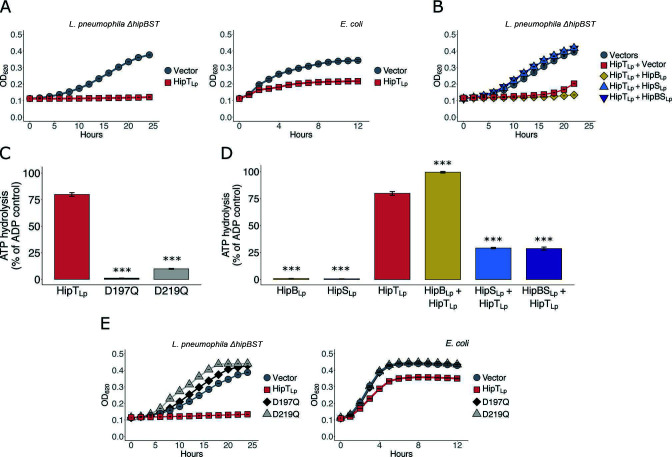
HipBST_Lp_ is a functional tripartite TA system that restricts growth via HipT_Lp_ kinase activity. (**A**) Expression of HipT_Lp_ in *L. pneumophila* cells with the endogenous *hipBST* locus deleted (∆*hipBST*) and *E. coli* (TOP10) cells. Expression was induced with IPTG (100 µM) for the pJB1806 expression vector used in *L. pneumophila* and arabinose (0.2%) for the pBAD18 vector used in *E. coli*. (**B**) HipT_Lp_ co-expression with HipB_Lp_ and HipS_Lp_ in *L. pneumophila* ∆*hipBST* cells. HipT_Lp_ was expressed from the pJB1806 vector, and HipB_Lp_ and HipS_Lp_ were co-expressed from the pNT562 vector. Expression was induced with IPTG (100 µM). (**C**) ADP-Glo kinase assay with purified recombinant His_6_-SBP-tagged HipT_Lp_. Both wild-type HipT_Lp_ and HipT_Lp_ with substitutions in two conserved catalytic residues (D197Q, D219Q) were assayed. Reactions contained 1 µg of protein and were incubated at 37°C for 30 min. (**D**) ADP-Glo kinase assay with purified recombinant His_6_-SBP-tagged HipB_Lp_, HipS_Lp_, and HipT_Lp_. Reactions contained 1 µg of each protein and were incubated at 37°C for 30 min. (**E**) Expression of HipT_Lp_ with mutations in two conserved catalytic residues (D197Q, D219Q) in *L. pneumophila* ∆*hipBST* and *E. coli* (TOP10) cells. Expression was induced with IPTG (100 µM) for the pJB1806 expression vector used in *L. pneumophila* and arabinose (0.2%) for the pBAD18 vector used in *E. coli*. All growth curves show the mean ± the standard deviation of three biological replicates. Data are representative of three independent experiments. All kinase assays show the mean ± the standard deviation of three technical replicates. Data are representative of a minimum of two independent experiments. Statistical hypothesis testing for the kinase assays was performed with a two-tailed Student’s *t*-test and each sample was compared to HipT_Lp_. ***P-value < 0.0001; α (0.05) was Bonferroni corrected for multiple hypothesis testing.

We next wondered whether the HipBST_Lp_ module could serve as a functional TA system. To test this, we cloned HipT_Lp_ into an inducible plasmid and examined the impact of its expression on bacterial growth. HipT_Lp_ expression inhibited growth in both an *L. pneumophila* strain with the endogenous locus deleted (∆*hipBST*) and *E. coli* (Fig. 3A; Fig. S3A). This effect was neutralized in *L. pneumophila* by the co-expression of HipS_Lp_ but not HipB_Lp_ (Fig. 3B; Fig. S3B). No growth inhibitory effects were observed for the expression of HipB_Lp_ or HipS_Lp_, either alone or in combination (Fig. S3C). These results are consistent with the reported functionality of the HipBST_Ec_ system (13) and confirm that HipBST_Lp_ is a functional TA module.

HipT_Ec_ has been shown to be a kinase and this activity is required for its toxicity to the cell (13). We, therefore, tested whether HipT_Lp_ also displayed kinase activity. We purified wild-type HipT_Lp_, HipT_Lp_(D197Q), and HipT_Lp_(D219Q) (Fig. S3D) and tested for ATP hydrolytic activity using the ADP-Glo kinase assay. Wild-type HipT_Lp_ demonstrated substantial activity, whereas the activity of both mutants was abrogated (Fig. 3C). ATP hydrolysis was also reduced for wild-type HipT_Lp_ by the addition of the kinase inhibitor 5′-fluorosulfonylbenzoyl-5′-adenosine (FSBA) (Fig. S3E). Co-incubation of purified recombinant HipS_Lp_ (Fig. S3D) with HipT_Lp_ neutralized activity *in vitro* (Fig. 3D), whereas co-incubation with HipB_Lp_ (Fig. S3D) did not and instead an increase in activity was observed. The cause of this is unclear, though it may be a consequence of promiscuous phosphorylation of HipB_Lp_ by HipT_Lp_. The D197Q and D219Q substitutions also eliminated the growth inhibitory phenotype of HipT_Lp_ in the ∆*hipBST* strain and *E. coli* (Fig. 3E; Fig. S3F and G), confirming that kinase activity is required for cellular toxicity. Taken together, these results demonstrate that HipBST_Lp_ is a functional TA system in which HipT_Lp_ toxicity results from its kinase activity, and neutralization is performed by HipS_Lp_ rather than HipB_Lp_.

### The HipBST_Lp_ system has the capacity for complex autoregulatory dynamics

**Fig 4:**
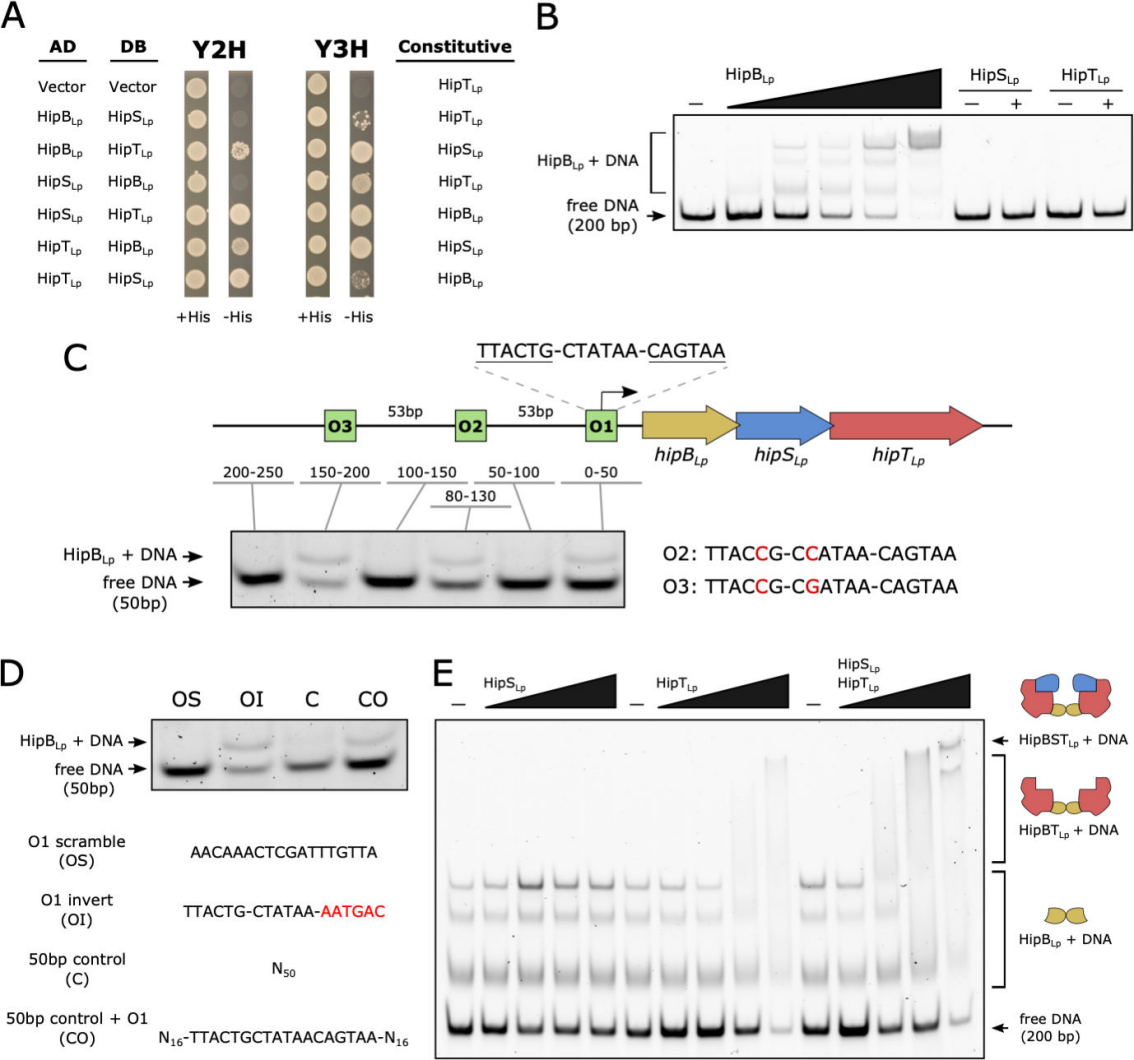
The HipBST_Lp_ system has the capacity for complex autoregulatory dynamics. (**A**) Y2H experiments testing for binary physical interactions in the HipBST_Lp_ tripartite proteins. Genes cloned into the pDEST-AD and pDEST-DB Y2H vectors are indicated, and representative images of *S. cerevisia*e Y8800 growth in the presence (+His) and absence (−His) of histidine are shown. Growth in the absence of histidine can only occur through a stable protein-protein interaction, due to the reconstitution of the GAL4 transcription factor (AD and DB domains) and subsequent expression of the *HIS3* reporter gene downstream of the *GAL1* promoter. Yeast two-hybrid experiments were also performed with a third protein constitutively expressed from the pAG416 vector (Y3H). Genes cloned into pAG416 are indicated. (**B**) Representative EMSA performed with recombinant purified HipBST_Lp_ proteins and a 200-bp DNA fragment (10 nM) encompassing the promoter region upstream of the *hipBST_Lp_* locus. HipB_Lp_ was added to DNA at concentrations of 1, 2.5, 5, 10, and 20 nM, and HipS_Lp_/HipT_Lp_ was added at a concentration of 20 nM. (**C**) Schematic of the operator sites upstream of *hipBST_Lp_* and consensus sequences for each site across 28 *Legionella* species. Nucleotide substitutions in O2 and O3 relative to O1 are colored red. Also displayed is a representative EMSA performed with 50-bp fragments from the 250-bp region upstream of *hipBST_Lp_*. HipB_Lp_ was added to DNA (10 nM) at a concentration of 2.5 nM. (**D**) Representative EMSA with 50-bp control DNA (10 nM) incubated with HipB_Lp_ (2.5 nM). Controls consisted of the 50-bp fragment containing O1 with a scrambled O1 sequence (OS), inversion of the downstream inverted repeat in O1 (OI; colored red), or a 50-bp random DNA control fragment (**C**) with the O1 sequence added (CO). The O1 sequence, where applicable, is displayed. (**E**) Representative EMSA showing increasing concentrations of HipS_Lp_ and HipT_Lp_ added to HipB_Lp_-DNA complexes (10 nM HipB_Lp_, 10 nM DNA). HipS_Lp_ and HipT_Lp_ were added at concentrations of 10, 40, 100, and 200 nM. All EMSA gels were stained with SYBR Green and protein-DNA complexes are indicated. Dashes indicate the absence of added protein.

Antitoxins of type II TA systems, such as HipB in the HipBA system, neutralize their cognate toxins via direct physical interaction (12). As HipS_Lp_ functions as the antitoxin in HipBST_Lp_, we wondered if it physically interacts with the HipT_Lp_ toxin. To test this, we examined the binary protein-protein interactions of the HipBST_Lp_ system using the yeast two-hybrid (Y2H) assay. Our results showed that HipT_Lp_ could physically interact with both HipB_Lp_ and HipS_Lp_ independently, but these two proteins could not themselves physically interact (Fig. 4A; Fig. S4A). To test whether all three proteins could stably associate, we repeated the experiment with the addition of constitutively expressed HipT_Lp_. In this setup, HipB_Lp_ and HipS_Lp_ came in close enough proximity to produce a detectable interaction (Fig. 4A; Fig. S4A), suggesting that HipT_Lp_ could interact with both proteins simultaneously and serve as a scaffold for a tripartite complex.

In the HipBA system, HipB both neutralizes HipA and regulates transcription of the *hipBA* locus (42). We next asked whether toxin neutralization and autoregulation of *hipBST_Lp_* transcription are decoupled, given the tripartite architecture of HipBST_Lp_. We tested all three proteins for the binding of 200-bp upstream (*hipBST_Lp_* promoter) sequence using the electrophoretic mobility shift assay (EMSA). Co-incubation with HipB_Lp_ slowed the migration of the DNA fragment (Fig. 4B), whereas HipS_Lp_ and HipT_Lp_ did not, indicating that transcriptional regulation of the *hipBST_Lp_* locus is indeed distinct from toxin neutralization. Interestingly, increasing concentrations of HipB_Lp_ produced a gel shift pattern that was absent when compared to two unrelated promoter controls, which showed only nonspecific DNA binding at higher concentrations (Fig. S4B).

Given these findings, we searched the *hipBST_Lp_* promoter region for putative DNA binding motifs and inverted repeats. This region does not contain either of the conserved operator sequences found in *hipBA* promoters (38, 43); however, we did detect an 18-nucleotide stretch containing an inverted repeat motif 24 nucleotides upstream of the HipB_Lp_ start codon (Fig. 4C). This sequence (referred to as O1) was almost perfectly conserved across all 28 *Legionella* species containing HipBST homologs (Fig. S4C) and overlaps with the predicted promoter of these systems. By comparing alignments of genomic sequence further upstream of *hipBST* systems, we detected two additional sites with asymmetrical inverted repeat sequences (termed O2 and O3) that are nearly identical to O1 except for two substitutions at the same position within both sequences (Fig. 4C; Fig. S4C). These core differences reflect the consensus sequences, however, and more variation is observed within O2 and O3 sequences across species relative to O1. The three putative operator sites are evenly spaced, with 53-bp inter-operator gaps in nearly all species, and no additional sites were detected further upstream or within the *hipB* gene sequence. To test whether HipB_Lp_ could bind each operator site, we performed EMSA experiments with 50-bp fragments of upstream DNA that either contained an operator site, did not contain one, or split one. Fragment migration was shifted only in the presence of a complete operator site (Fig. 4C), regardless of HipB_Lp_ concentration (Fig. S4D). We next examined HipB_Lp_ specificity for the O1 operator site—as it was the most conserved—by mutating the 50-bp fragment containing O1 to either completely scramble the O1 sequence or abolish its inverted repeat symmetry. Scrambling O1 was sufficient to abrogate the shift in fragment migration; however, this shift was still observed with abolished inverted repeat symmetry (Fig. 4D; Fig. S4E). Finally, we tested two additional 50-bp fragments containing either random 50-bp sequence or random sequence flanking an insertion of the O1 site. From this, we observed that the presence of the O1 site resulted in improved fragment shift efficiency in a concentration-dependent manner (Fig. 4D; Fig. S4E). In summary, we identified a conserved set of operator sites within the upstream promoter region of *hipBST* systems that are both necessary and sufficient for HipB binding.

We next wondered what effect co-incubation with HipS_Lp_ and HipT_Lp_ would have on the HipB_Lp_ binding of promoter DNA. To address this, we performed EMSAs with increasing concentrations of either protein alone or together added to the HipB_Lp_-DNA complex. The addition of HipS_Lp_ to HipB_Lp_ had no impact on DNA migration, regardless of concentration, whereas co-incubation with HipT_Lp_ produced a secondary gel shift indicative of the formation of a HipT_Lp_-HipB_Lp_-DNA complex (Fig. 4E; Fig. S4F). Furthermore, the co-incubation of HipB_Lp_ with HipS_Lp_ and HipT_Lp_ resulted in a secondary gel shift relative to HipT_Lp_ alone. This additional shift in the presence of all three proteins also occurred at a lower concentration of HipS_Lp_/HipT_Lp_ relative to HipT_Lp_ alone (Fig. 4E; Fig. S4F). Interestingly, the highest stoichiometric excess of either HipT_Lp_ or HipS_Lp_/HipT_Lp_ relative to HipB_Lp_ also appeared to result in a decrease in unbound DNA in the reaction. These results demonstrate the *in vitro* formation of HipB_Lp_-DNA and HipT_Lp_-HipB_Lp_-DNA complexes and are consistent with the formation of a HipS_Lp_-HipT_Lp_-HipB_Lp_-DNA complex. Taken together, our findings reveal the potential for multiple binary and ternary interactions in the HipBST_Lp_ system and provide insights that distinguish transcriptional autoregulation by HipB_Lp_ from other HipB proteins. HipB_Ec_ has also been reported to regulate the transcription of the *hipBST_Ec_* locus (44); however, our data demonstrate the occurrence of a HipB_Lp_-HipT_Lp_-DNA complex and suggest that interaction dynamics between binary and ternary protein combinations may serve as additional layers in a larger regulatory program.

### HipBST_Lp_ neutralization exploits the P-loop ejection mechanism of HipA_Ec_

**Fig 5:**
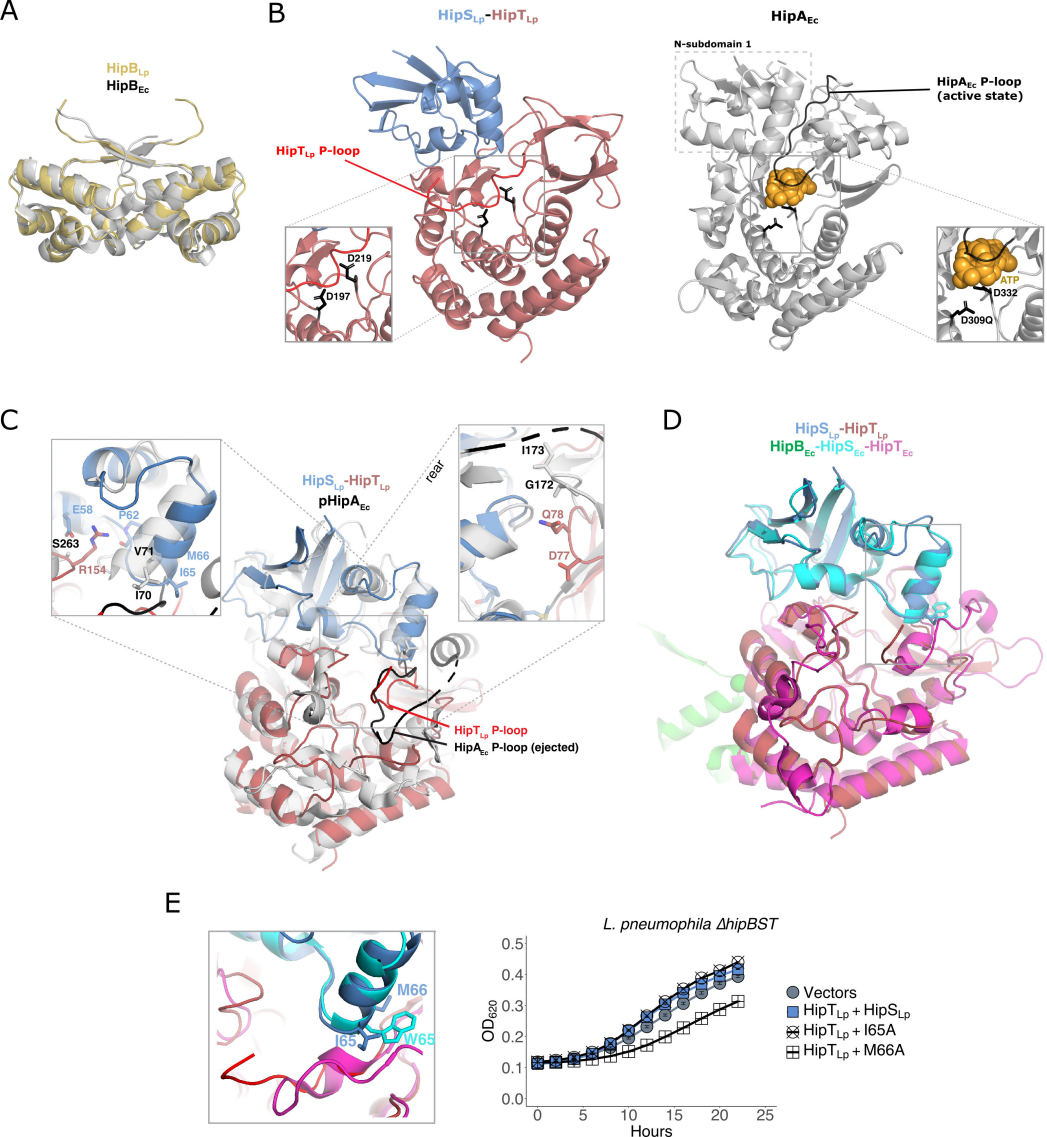
HipT_Lp_ neutralization by HipS_Lp_ utilizes a P-loop ejection mechanism. (**A**) Alignment of the HipB_Lp_ structure with HipB_Ec_ (4YG1). (**B**) Comparison of the HipS_Lp_-HipT_Lp_ co-crystal structure with HipA_Ec_ (3DNT) in which the HipA_Ec_ P-loop motif is in the internalized and active conformation. The HipA_Ec_ N-subdomain 1, corresponding to HipS_Lp_, is indicated with a dashed box. The P-loops of both HipT_Lp_ (red) and HipA_Ec_ (black) are indicated. ATP in the HipA_Ec_ catalytic pocket is depicted with yellow spheres. Insets display the catalytic pocket of both kinases, along with two conserved residues involved in ATP coordination (D197, D309Q) and Mg^2+^ binding (D219, D332). (**C**) Alignment of the HipS_Lp_-HipT_Lp_ structure and pHipA_Ec_ (3TPE), in which the P-loop motif is autophosphorylated and in the inactive, ejected conformation. Insets display two regions of variation between the HipS_Lp_-HipT_Lp_ interaction interface and HipA_Ec_: a shifted helix in HipS_Lp_ (inset on the left) and a loop in the N-terminal region of HipT_Lp_ (inset on the right). Key residues in each region are labeled. (**D**) Alignment of the HipS_Lp_-HipT_Lp_ structure with the structure of HipBST_Ec_ (7AB5). (**E**) Left: inset from (**F**) comparing the tryptophan residue in HipS_Ec_ (W65) with the methionine (M66) and isoleucine (I65) residues at the same position in HipS_Lp_. Right: co-expression of HipT_Lp_ (pJB1806) with wild-type or mutant (I65A, M66A) HipS_Lp_ (pNT562) in *L. pneumophila* ∆*hipBST* cells. Expression was induced with IPTG (100 µM). Growth curves show the mean ± the standard deviation of three biological replicates. Data are representative of three independent experiments.

To understand the structural basis for neutralization in the HipBST_Lp_ system, we solved the *apo* structure of HipB_Lp_ and the co-crystal structure of HipS_Lp_-HipT_Lp_ (Table S7). HipB_Lp_ is highly similar to HipB_Ec_ (RMSD 1.6–2.2 Å over approximately 70 Cɑ atoms) and adopts a dimeric helix-turn-helix conformation, consistent with its capacity to bind DNA (Fig. 5A). Strikingly, the structural conformation of the HipS_Lp_-HipT_Lp_ complex is nearly identical to HipA_Ec_ (RMSD 2.8 Å over 421 Cɑ atoms, when considering HipS_Lp_ and HipT_Lp_ as a single chain) (Fig. 5B), despite the functional divergence between the systems. Given that HipS_Lp_ serves the role of antitoxin, we wondered how neutralization could be achieved in this orientation. To address this, we compared the HipS_Lp_-HipT_Lp_ structure to previously solved HipA_Ec_ structures. Independent of neutralization by HipB_Ec_, HipA_Ec_ is capable of self-inhibition through intermolecular phosphorylation of its S150 residue (45). This site is found within the catalytic P-loop, and its phosphorylation or mutation to alanine yields a conformational shift whereby the P-loop becomes ejected and solvent exposed, thereby inactivating the toxin (Fig. 5C). Part of the P-loop in our HipS_Lp_-HipT_Lp_ structure was unresolved—suggesting it was in a flexible or dynamic state—but the resolved portion (residues 50–56) was oriented in a manner highly similar to the ejected and inactive HipA_Ec_ P-loop (Fig. 5C). This suggests that the interaction between HipS_Lp_ and HipT_Lp_ facilitates P-loop ejection to neutralize the toxin. During our analyses, the structures of both HipT_Lp_ and HipS_Lp_-HipT_Lp_ were reported by another group (21). These illustrate the orientation of the P-loop in its internalized and catalytically active state, and as in our HipS_Lp_-HipT_Lp_ structure, this motif becomes unresolved and likely ejected upon HipS_Lp_ binding. In support of this, both HipS_Lp_-HipT_Lp_ co-crystal structures show a high degree of concordance (HipS: RMSD 0.25 Å and TM-score 0.99 over 101 Cɑ atoms; HipT RMSD 0.25 Å and TM-score 0.96 over 261 Cɑ atoms) and are nearly identical in orientation, even with regard to the resolved portion of the P-loop. These findings are consistent with similar structural observations in the HipBST_Ec_ system (44), suggesting a conserved P-loop ejection model of HipBST neutralization.

To better understand the evolution of this unique mechanism, we sought to identify sites of divergence between the HipS_Lp_-HipT_Lp_ complex and HipA_Ec_. Two major conformational changes are present in HipT_Lp_ loop 75–80 and HipS_Lp_ helix 65–66, relative to the equivalent regions in HipA_Ec_ (171–172 and 70–71), which shift these regions inward and impinge on the space where the P-loop would be internalized in its active state (Fig. 5C). We identified two residues in HipT_Lp_ (D77, Q78) that occupy this space and are not conserved in HipA_Ec_ (Fig. S1A). Substitution of either residue with alanine did not impair HipT_Lp_ toxicity (Fig. S5A); however, it also did not prevent neutralization by HipS_Lp_ (Fig. S5B). The HipT_Lp_ residue R154 is also not conserved in HipA_Ec_ or HipT_Ec_ and appears to contribute to the interaction between toxin and antitoxin through the formation of a hydrogen bond to the backbone of HipS_Lp_(P62) and a salt bridge with HipS_Lp_(E58) (Fig. 5C). However, mutation of this residue to alanine (R154A) was again not sufficient to impair the neutralization of HipT_Lp_ by HipS_Lp_ (Fig. S5A and B).

### HipT_Lp_ neutralization by HipS_Lp_ does not rely on the tryptophan residue utilized by HipS_Ec_

We next compared the *L. pneumophila* and *E. coli* HipBST systems directly, as a structure of HipBST_Ec_ was recently reported (44) (Fig. 5D). In the HipBST_Ec_ system, neutralization has been found to depend on a tryptophan residue in HipS_Ec_ (W65), which projects into the P-loop containing pocket of HipT_Ec_ (Fig. 5D and E). This residue appears critical for HipS_Ec_ function and is conserved across numerous HipS_Ec_ homologs, but is absent from HipS_Lp_ and instead, this site is occupied by much smaller methionine and isoleucine residues (I65, M66). We substituted both residues with alanine and observed a partial reduction in HipT_Lp_ neutralization by HipS_Lp_(M66A), but no effect for HipS_Lp_(I65A) (Fig. 5E; Fig. S5C). Consistent with these results, neither substitution impaired the physical interaction between HipS_Lp_ and HipT_Lp_ (Fig. S5D). In summary, the HipS_Lp_-HipT_Lp_ P-loop ejection mechanism is broadly conserved with HipBST_Ec_, yet is achieved through different motifs and interactions within the toxin-antitoxin interface.

### HipT_Lp_ toxicity is not inhibited by autophosphorylation of its P-loop serine residue

The HipBST_Ec_ system was recently shown to contain a double serine motif in the HipT_Ec_ P-loop (S^57^IS^59^), which is proposed to allow for a dual autoregulatory dynamic in that system (44). This motif is absent from HipT_Lp_, which contains only a single serine in this position (S54), similar to HipA_Ec_ (S150) (Fig. S1A). While many of the previously reported HipT homologs from the Gammaproteobacteria (24) contain either an SxS motif or some combination of double S/T residues, the corresponding motif in HipT_Lp_ (SVQ) is absent in these homologs but is either conserved or nearly identical (SIQ) across the HipT_Lp_ homologs in *Legionella* species. It has been shown previously that autophosphorylation or substitution of the single P-loop serine in HipA_Ec_ leads to loss of activity (40, 45). We therefore wondered what consequence modifying this residue would have on HipT_Lp_ toxicity. To test this, we constructed mutations that both ablate (S54A) and mimic (S54D) phosphorylation at this position. Neither mutation inhibited toxicity (Fig. S5E), which was surprising given that the corresponding mutation in HipT_Ec_ (S57A) renders the protein non-toxic (44), as does the S150A mutation in HipA_Ec_ (40), and autophosphorylation of HipA_Ec_ leaves it unable to bind ATP and retain catalytic activity (45). These findings are supported by recent structural work demonstrating that HipT_Lp_ can bind ATP despite being autophosphorylated (21) and highlight an unusual autoregulatory difference between these systems.

### The cellular target of HipT_Lp_ is different from those of characterized HipT and HipA toxins

**Fig 6:**
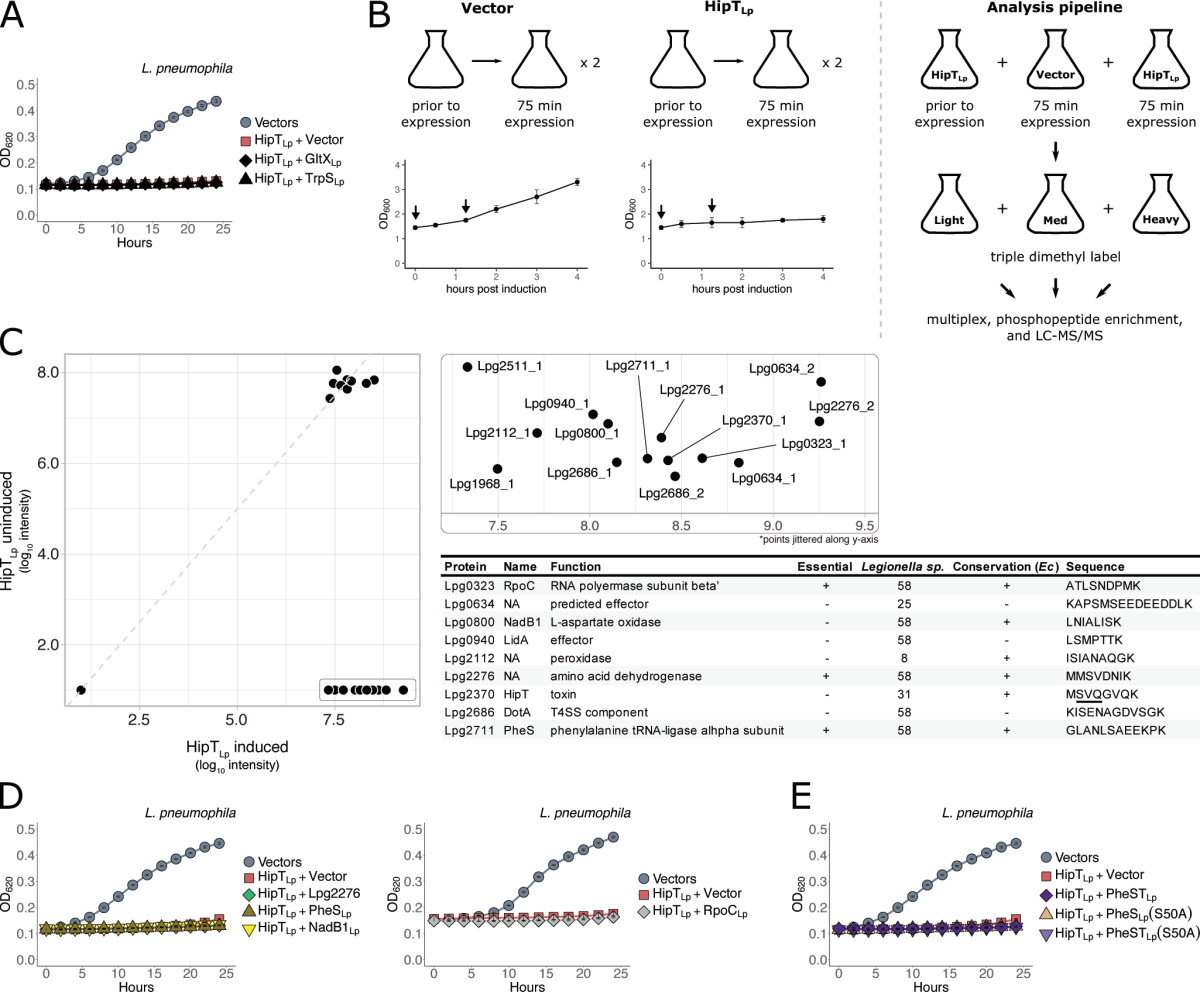
HipT_Lp_ has an unknown cellular target that is not conserved with characterized HipT or HipA toxins. (**A**) Co-expression of HipT_Lp_ (pJB1806) with GltX_Lp_ or TrpS_Lp_ (pNT562) in *L. pneumophila* cells. (**B**) Overview of phosphoproteomic experimental design. Left: cultures of *L. pneumophila* ∆*hipBST* carrying either pJB1806 or pJB1806::*hipT*_*Lp*_ were grown to mid-log phase in the presence of 0.5% glucose, after which expression was induced by the addition of IPTG (100 µM). Prior to and 75 min postinduction (indicated by arrows on growth curves), cells were harvested for phosphoproteomic analysis. This experiment was performed independently twice, and growth curves show the mean ± the standard deviation from the two independent experiments. Right: cell lysates from cultures expressing HipT_Lp_ at both time points, and the vector control postinduction, were dimethyl labeled and multiplexed, enriched for phosphopeptides, and analyzed by mass spectrometry. The HipT_Lp_-uninduced, vector-induced, and HipT_Lp_-induced samples were labeled with the light, intermediate (med), and heavy channels, respectively. (**C**) Phosphopeptides detected in both HipT_Lp_-uninduced and induced samples (left) and those enriched during HipT_Lp_ expression only (inset) are displayed. The dashed gray line is *y* = *x* for comparison between channels. In the inset, the suffix denotes the peptide number seen from a given ORF. The intermediate (vector-induced) channel is excluded from this plot for simplicity and because the results did not change the list of candidate substrates. The table contains candidate phosphoproteins that were observed across both replicates (excludes Lpg1968 and Lpg2511) along with their function, essentiality in broth, conservation in 58 *Legionella* species and *E. coli* (*Ec*), and detected phosphopeptide sequence. Candidates chosen for subsequent validation are highlighted in gray. The HipT_Lp_ phosphosite is underlined. (**D**) *L. pneumophila* cells co-expressing HipT_Lp_ (pJB1806) with phosphoproteomic candidates (pNT562) that were either essential or highly conserved in both *Legionella* and conserved in *E. coli*. (**E**) Co-expression of HipT_Lp_ with PheST_Lp_ or phosphomimetic mutants of PheS_Lp_(S50A) in *L. pneumophila* cells. Growth curves show the mean ± the standard deviation of three biological replicates. Expression was induced using IPTG (100 µM) for all constructs. Data are representative of three independent experiments.

Despite their phylogenetic divergence, the HipBST_Lp_ system functions similarly to HipBST_Ec_. Given the dearth of comparisons between TA homologs across distantly related bacteria, we chose to further explore the functional conservation between systems by testing whether they modified the same substrate. HipT_Ec_ was previously shown to phosphorylate tryptophan tRNA-ligase (TrpS) in order to arrest cellular growth (13), whereas the canonical target of HipA_Ec_ is glutamyl tRNA-synthetase (GltX) (46, 47). TrpS and lysine tRNA-ligase have also been reported as substrates for the HipBA systems in *Caulobacter crescentus* (15, 48). In *E. coli*, co-expression of TrpS or GltX with HipT_Ec_ or HipA_Ec_, respectively, rescues the growth inhibitory effect of the toxins. In fact, *E. coli* TrpS was sufficient to rescue the toxicity of HipT homologs from *Haemophilus influenzae* and *Tolumonas auensis* as well (13). We therefore tested whether TrpS_Lp_ or GltX_Lp_ could rescue HipT_Lp_-induced growth inhibition when co-expressed in *L. pneumophila*. Surprisingly, neither of these proteins could alleviate HipT_Lp_ toxicity (Fig. 6A; Fig. S6A), suggesting that HipT_Lp_ poisons the cell by targeting one or more previously undescribed substrates.

GltX and TrpS were originally identified as the targets of HipA_Ec_ and HipT_Ec_ by screening *E. coli* genomic libraries for genes whose overexpression rescued toxin-induced growth inhibition (13, 46) or cold sensitivity (47). Given the observed toxicity of HipT_Lp_ in *E. coli* cells, we used the same approach to search for putative targets. We pooled and transformed the *E. coli* ASKA library into BL21 cells expressing HipT_Lp_ (pCDF1-b) and screened the resulting transformants. We observed robust growth inhibition upon HipT_Lp_ expression using an empty vector control (pCA24N), further demonstrating the toxicity of HipT_Lp_ in *E. coli* (Fig. S6B). When cells were transformed with the ASKA library pool, we recovered a small number of transformants across multiple screen replicates (Fig. S6B). Despite several clones exhibiting a stable growth rescue phenotype, sequencing revealed no clear enrichment of any genes or pathways, and subsequent attempts at validation in *L. pneumophila* were unsuccessful.

As rescue screening of the ASKA library did not produce any strong candidate HipT_Lp_ targets, we next pursued an orthogonal approach. Phosphoproteomic analysis was used previously to identify TrpS as a substrate of *C. crescentus* HipA2 (48) and to demonstrate the enrichment of GltX phosphorylation in the HipA_Ec_ phosphoproteome (49). We therefore performed a phosphoproteomic screen in *L. pneumophila* to detect substrates that were only modified under conditions of HipT_Lp_ overexpression, relative to both uninduced HipT_Lp_ and empty vector control (Fig. 6B). From this, we identified a small number of proteins that were enriched for phosphopeptides during HipT_Lp_-induced growth inhibition (Fig. 6C; Table S8). We also observed that HipT_Lp_ is phosphorylated on S54, thereby demonstrating the occurrence of this modification *in vivo*. Of the eight candidate substrates identified, three are known to be essential for growth (50, 51), six are conserved across all *Legionella* species, and five have orthologs in *E. coli* (Fig. 6C). While HipA_Ec_ has been shown to phosphorylate a large pool of proteins, only co-expression with GltX is sufficient to rescue its growth inhibitory phenotype (49). In order to validate our candidate substrates, we cloned a subset of hits that were either essential or highly conserved and tested them by co-expression with HipT_Lp_. Under these conditions, no putative substrate was able to rescue growth inhibition in *L. pneumophila* (Fig. 6D; Fig. S6C).

One of the hits we detected was the alpha subunit (PheS) of phenylalanine tRNA-ligase. Given that both GltX and TrpS are tRNA-ligases, this class of target would be consistent with other Hip toxins. Interestingly, phenylalanine tRNA-ligase is composed of two separate subunits, whereas GltX and TrpS are both single proteins. To test whether the complete phenylalanine tRNA-ligase (PheST_Lp_) was required for growth rescue, we co-expressed it with HipT_Lp_ in *L. pneumophila*. However, we did not observe any rescue of growth inhibition (Fig. 6E; Fig. S6D). From our phosphoproteomic data, we also observed that PheS was phosphorylated on the S50 residue during HipT_Lp_ overexpression. We next tested whether mutation of this site to ablate phosphorylation (S50A) would prevent growth inhibition; however, neither PheS(S50A) nor PheST(S50A) was able to rescue *L. pneumophila* growth when co-expressed with HipT_Lp_ (Fig. 6E; Fig. S6D). While these approaches were unable to identify a definitive toxic substrate of HipT_Lp_, they nevertheless suggest that its cellular target is likely not conserved with HipT_Ec_ or any characterized HipA homologs.

## DISCUSSION

The ubiquity of toxin-antitoxin systems in bacterial genomes and their capacity for selfish maintenance have provided strong evidence for their role as parasitic elements. Given the plastic nature of the accessory genome and the patchy distribution of TA systems, the occurrence of highly conserved elements would suggest selection beyond mere addiction. Here, we characterize a HipBST system in *L. pneumophila*, which is highly conserved across *Legionella* species, despite the genomic variation within this genus. Indeed, more than half of the *Legionella* species we searched have either a complete HipBST module (28/58) or individual HipBST genes (8/58). This level of conservation is notable, in that it is higher than both the average accessory gene (26/58) and *L. pneumophila* effector (16/58).

In characterizing HipBST_Lp_, we detected a large number of closely related homologs across diverse bacterial taxa. This greatly expands the sequence space of HipBST systems and reveals considerable diversity within this newly described TA family. By comparing the individual protein phylogenies (Fig. S1C), we observed an interesting pattern whereby HipB sequences from HipBA_So_-like systems cluster with HipB sequences from HipBST systems. The structural similarities between HipBA_So_ and HipBST_Ec_ have been noted previously (44) and given the difference in HipB orientation between these systems and HipBA_Ec_, this could suggest an evolutionary relationship between HipBA_So_ and the newly discovered HipBST systems.

We were surprised to discover that homologs of HipBST_Lp_ are almost entirely restricted to the *Legionella* genus within the Gammaproteobacteria but are widely distributed in other taxonomic groups. This could suggest a shared environmental niche, common functional role, or frequent DNA exchange between these taxa and Legionellales. Previous work has only compared HipBST systems that are closely related to HipBST_Ec_ (13), and as the sequence space of HipBST_Lp_ homologs appears to be both more diverse and taxonomically distributed (Fig. 1C and D), this offers the potential to reveal even greater breadth of TA biology within this family. One pattern that emerges from our initial data is an apparent exclusion between different HipBST clades (Fig. 1D). While it is tempting to speculate that some difference in biological function (e.g., persistence or chromosomal stabilization) or cellular target could be responsible for the taxonomic distribution of these two system types, it is important to note that further work is required to confidently establish this pattern. Importantly, our analyses focused on closely related rather than remote homologs of each TA system, and thus do not incorporate distant homologs that would be otherwise detected using more sensitive search methods or highly divergent systems comprising components from multiple system types. This was done to examine the taxonomic distribution of systems with possible shared ancestry and evolutionary history, and because our findings suggest that remote homology (such as between HipBST_Ec_ and HipBST_Lp_) may mask important biological differences. However, we do note this methodological limitation of our analyses, and because of this, we cannot make assertions about the taxonomic distribution patterns across more distantly related HipBST homologs.

During our genomic search, we discovered a putative duplicate HipBST system downstream of HipBST_Lp_. This locus (*lpg2377-80*) encodes five predicted ORFs and is only found in the *L. pneumophila* genome (Fig. S7A). Its unusual architecture is achieved through the splitting of both *hipS* and *hipT*, resulting in the separation of the P-loop and kinase core in HipT. Interestingly, all catalytically critical motifs remain conserved in split-HipT (Fig. S7A). To determine whether this system was functional, we expressed both split-HipT_Lp_ and split-HipS_Lp_ in *L. pneumophila* cells. Despite the preservation of the P-loop motif, the expression of either split protein pair, alone or in combination, did not exhibit a growth inhibitory effect (Fig. S7B). We also observed a similar organization in the HipBST system encoded by *Legionella gormanii*, where *hipT* is again split, but in this case, the upstream ORF is truncated due to a frameshift and the catalytic P-loop is no longer intact (Fig. S7C). Thus, this system appears to be in a state of decay or functional divergence. While it remains to be seen how these split-protein architectures affect system functionality, their growing diversity attests to the substantial modularity and evolvability of the Hip TA systems.

A striking feature of the HipBST_Lp_ system is the previous claim that HipT_Lp_ is an *L. pneumophila* effector (20, 21). This would represent an exceedingly rare example of toxin-antitoxin system/eukaryotic effector bifunctionality, as there is limited evidence of TA modules being repurposed as interdomain translocated substrates (7). However, we found no evidence of HipT_Lp_ translocation beyond levels of the negative control FabI. Instead, the broad taxonomic distribution of HipBST_Lp_ homologs likely extends to many species that do not deliver effectors into host cells, further arguing against this functionality. HipT_Lp_ was first hypothesized to be an effector due to the presence of a putative translocation motif (52); however, re-examination determined it was unlikely to encode a true secretion signal (53). Our inability to observe HipT_Lp_ translocation above control levels, therefore, argues for caution in ascribing functionality to HipT_Lp_ within the eukaryotic host. Given that cytosolic proteins (such as FabI) can be translocated at low frequencies (39, 54) or their localization potentially influenced by bacterial cell lysis events, basal level of secretion should be confirmed with orthologous methodologies, inactive mutants unlikely to cause cell lysis, careful quantification, and several controls. Incontrovertible evidence for HipT_Lp_ effector activity may come from the phenotypic observation of an impact on the host cell during infection. In the absence of this, exceeding a threshold ratio of quantified fluorescence is the necessary standard for effector validation (39, 55).

**Fig 7:**
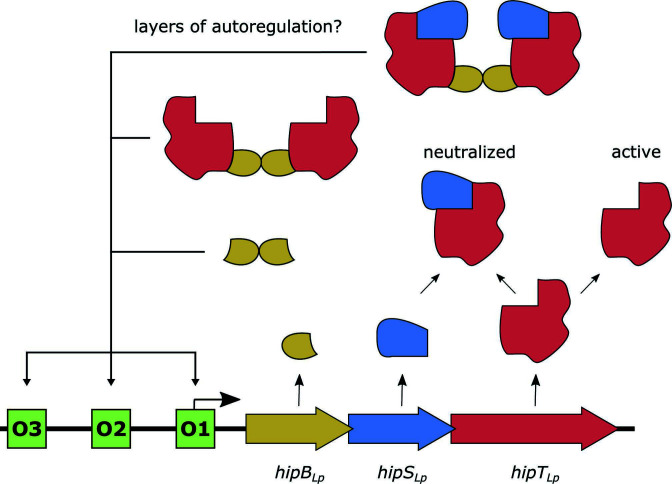
Autoregulatory capacity of the HipBST_Lp_ toxin-antitoxin system. HipT_Lp_ is neutralized by physical interaction with HipS_Lp_ and stable interactions are additionally observed between HipB_Lp_-HipT_Lp_ and HipB_Lp_-HipS_Lp_-HipT_Lp_. HipB_Lp_ can bind promoter DNA at three conserved operator sites upstream of *hipBST_Lp_* to regulate system expression and these sites contain unique sequence motifs relative to HipBA systems. Protein-DNA complexes formed by HipB_Lp_-DNA, HipT_Lp_-HipB_Lp_-DNA, and HipS_Lp_-HipT_Lp_-HipB_Lp_-DNA suggest the possibility for multiple layers of transcriptional regulation in the system that could be influenced by relative protein stoichiometry and complex stability.

In this work, we demonstrate that HipBST_Lp_ is a TA system—the core functionality of which is conserved with HipBST_Ec_. A recent report on HipBST_Lp_ (21) included the observation that HipT_Lp_ does not inhibit growth in *E. coli*. We routinely observed that overexpression of HipT_Lp_ inhibits *E. coli* growth, using multiple strains, vectors, and conditions (Fig. 3A; Fig. S3F, G, and S6B). Our detection of a physical interaction between HipB_Lp_ and HipT_Lp_, by both yeast two-hybrid and EMSA assays, is also contradictory to the previous report (21), which found no such interaction. The absence of an interaction between HipB_Lp_ and HipT_Lp_ would suggest that only the neutralized HipS_Lp_-HipT_Lp_ complex would participate in the autoregulatory activity of HipB_Lp_. Instead, our observations are consistent with the formation of HipB_Lp_-DNA, HipT_Lp_-HipB_Lp_-DNA, and HipS_Lp_-HipT_Lp_-HipB_Lp_-DNA complexes (Fig. 4E; Fig. S4F). This raises the possibility of multi-layered regulation in the HipBST_Lp_ system, whereby dimeric, binary, and ternary protein interactions could influence system expression (Fig. 7). For example, the tripartite protein interaction dynamics may influence HipB_Lp_ binding affinity for promoter DNA or produce differential stability of protein-DNA complexes as a feedback mechanism for system repression/derepression. An emphasis of future work should, therefore, be to investigate the changes in HipB_Lp_-DNA interactions and affinity in the presence of HipS_Lp_ and HipT_Lp_, in addition to examining what impact, if any, the phosphorylation state of HipT_Lp_ has on these interactions (38).

While investigating the regulatory dynamics of HipBST_Lp_, we discovered and characterized three conserved operator sites upstream of *hipBST* homologs across *Legionella* species (Fig. 7). The presence of three sites is concordant with the gel shift patterns we observed when incubating HipB_Lp_ with promoter DNA (Fig. 4B) and raises the intriguing possibility that HipB_Lp_-DNA complexes with intermediate occupancy can be formed, in contrast to the cooperativity observed with HipB from HipBA_Ec_ (42). These are the first operators identified for a HipBST TA system and are notable in that they differ in both their inverted repeat motifs and overall structure relative to previously identified HipBA operators (38, 43). The *Legionella* HipBST inverted repeat sequences are composed of six nucleotides rather than the five in HipBA systems, possess larger inter-operator spacer distances, and the sequence between inverted repeats is largely conserved across operators rather than being variable. Importantly, O2 and O3 contain single nucleotide substitutions that abolish inverted repeat symmetry, yet are still bound by HipB_Lp_ in a concentration-dependent manner comparable with O1. This variation between operator sites within the same genome is, therefore, a unique aspect of HipBST autoregulation, with one possible explanation being that HipB binds each site with differing affinity. We did not observe HipB_Lp_ affinity differences in our EMSA assays (Fig. S4D); however, future work should address this and the potential for intermediate occupancy protein-DNA complexes with more sensitive methodologies, in addition to identifying which HipB_Lp_ residues and operator sites influence DNA binding and specificity. Interestingly, the HipBST operator motifs we identified are absent from the promoter region upstream of *hipBST_Ec_*, which further supports the functional divergence between these two systems. We were not able to detect these motifs at other sites throughout the *L. pneumophila* genome, except for upstream of the pentapartite system, which is consistent with a putative duplication event for this locus.

We were surprised to find that the HipS_Lp_-HipT_Lp_ complex adopts a conformation that is almost unchanged from that of HipA_Ec_, consistent with recently reported structures for both HipBST_Ec_ (44) and HipBST_Lp_ (21). Neutralization in both HipBST systems is achieved by exploiting an intrinsic property of HipA biology—namely its P-loop ejection mechanism of autoregulation. The HipBST_Ec_ and HipBST_Lp_ systems achieve this functionality through shared structural motifs, while also displaying several key differences. In particular, while the bulky W65 residue in HipS_Ec_ is both critical for toxin neutralization and conserved in many other HipS_Ec_ homologs (44), in HipS_Lp_ this position is occupied by the much smaller I65 and M66 residues. These do not impinge on the catalytic pocket of HipT_Lp_ to the same extent as W65 and are not necessary for HipS_Lp_-HipT_Lp_ physical interaction or neutralization. The absence of an analogous residue to W65 in HipS_Lp_, therefore indicates alternate biochemical means of achieving P-loop ejection may be present across HipBST system homologs.

A double serine motif (S^57^IS^59^) was recently characterized in HipT_Ec_ (44), with the differential autophosphorylation of either serine reported to affect toxin neutralization and activity. This motif is absent from HipT_Lp_, which instead contains a single P-loop serine (S54) similar to the S150 residue in HipA_Ec_. We observed that the phosphorylation state, or substitution of this residue with alanine, does not affect protein toxicity. This was unexpected, given that both pHipA_Ec_ and HipA_Ec_(S150A) cannot bind ATP and are not toxic (45). In support of this unusual behavior, a structure of pHipT_Lp_ bound to an ATP analog was recently reported (21), further demonstrating the retention of HipT_Lp_ activity despite its phosphorylation state. Why HipT_Lp_ autoregulation differs in such a critical aspect from HipA_Ec_ is an outstanding question, as this would seemingly prohibit cell detoxification via *trans* autophosphorylation. Phosphomimetic mutation of either serine residue in HipT_Ec_ (S57D, S59D) does not inhibit toxicity (44); however, mutation of either residue to alanine appears to abrogate activity—though conflicting results have been reported (21). Regardless, our findings demonstrate that the autophosphorylation state of HipT_Lp_ is not required for its activity, indicating a shift in the autoregulatory functionality of the HipT_Lp_ kinase.

The most critical difference we observed between HipBST_Ec_ and HipBST_Lp_ is the failure of either GltX_Lp_ or TrpS_Lp_ to rescue HipT_Lp_ toxicity. This was further supported by the absence of both GltX_Lp_ and TrpS_Lp_ in our genomic rescue and phosphoproteomic screens. We did, however, observe a small set of candidate proteins that were only phosphorylated during HipT_Lp_ overexpression in *L. pneumophila* cells. In particular, we detected the phosphorylation of PheS, which is also a tRNA-ligase like GltX and TrpS, and thus a strong candidate for the target of HipT_Lp_. While none of the putative targets were sufficient to rescue *L. pneumophila* growth when co-expressed with HipT_Lp_, this does not eliminate them from consideration. Instead, this may be a consequence of some aspect of *L. pneumophila* or HipT_Lp_ biology that is not conducive to kinase target saturation for growth rescue, or possibly the requirement of multiple co-expressed substrates to overcome toxin activity. For example, while we observed no rescue upon co-expressing one promising phosphorylated candidate–the β′-subunit of RNA polymerase (RpoC)–with HipT_Lp_, the complete enzyme is composed of three additional subunits (RpoA, RpoB, and RpoZ). It is possible that co-expression of each of these four subunits in stoichiometric amounts may be required to rescue growth inhibition by HipT_Lp_. An aim of future work will, therefore, be to further test the candidate targets identified using alternative means of validation, in addition to co-expression tests that include functional interactors, such as the reconstitution of the entire RNA polymerase enzyme. In light of our results, it should also be considered whether HipT_Lp_ in fact phosphorylates a protein at all to poison the cell, as the true target may be a nucleic acid or other molecule. Conversely, the presence of both DotA and multiple effectors in our phosphoproteomic data may reflect either a previously undescribed mode of toxicity, such as disrupting the translocation apparatus (which is known to have effects on axenic growth) (56) or reveal a regulatory functionality of HipT_Lp_ with regard to effector secretion. As such, future work should focus on first identifying the overarching biological process (such as macromolecular biosynthesis), if any, that HipT_Lp_ perturbs. One approach might be to utilize metabolic labeling assays that monitor the incorporation of radiolabeled methionine, uridine, and thymidine, in order to determine whether HipT_Lp_ expression has a differential effect on rates of translation, transcription, or DNA synthesis, respectively—as has been found with other toxins (57, 58). Were this to be inconclusive, alternative strategies such as suppression mutant or gene knockout library screening could also be undertaken, in addition to testing the effect of HipT_Lp_ on Dot/Icm assembly or function. Regardless, we report that HipT_Lp_ likely has a different cellular substrate from characterized HipT and HipA toxins, further demonstrating its functional divergence from HipT_Ec_.

Given the conservation of HipBST homologs across *Legionella* species, we wondered whether this system showed any pattern of association with chromosomal subregions or signatures of acquisition. The genome of *L. pneumophila* is organized into discrete clusters of essential and non-essential genes—the latter of which are enriched for effectors and mostly dispensable for broth growth and host infection (50, 51). The HipBST_Lp_ locus is found within one such cluster (Fig. S8A), and this led us to hypothesize that other HipBST_Lp_ systems might also be found within accessory genomic regions of non-essential genes. To test this, we predicted regions of non-essentiality across the 28 *Legionella* species’ genomes with HipBST systems. We found that HipBST is frequently associated with non-essential gene clusters (22/28) and that neither the local genetic neighborhood nor broader cluster composition is well conserved across HipBST_Lp_-containing clusters (Fig. S8B). We further extended this search to incorporate remote homologs in the gene neighborhood of HipBST_Lp_ (Fig. S8C); however, despite the increased search sensitivity, we observed relatively poor conservation of most proximal elements (≤30% of clusters). Interestingly, one gene neighbor (WP_015444114.1) did co-occur with the majority of HipBST systems (75%) and was predicted to encode either a recombinase or integrase, which could indicate a possible origin for HipBST acquisition with *Legionella* species. The high conservation of HipBST modules, contrasted with the low overall cluster conservation, raises the possibility that these modules may serve to stabilize their unique genomic neighborhood in each species. Indeed, tripartite TA systems are more commonly found on mobile genetic elements and have been hypothesized to function in DNA stabilization (12). This would be advantageous for *Legionella* species, which encode vast and diverse effector repertoires—including some with fitness costs in specific hosts (51, 59). Given the broad host range of *Legionella*, maintaining effector diversity likely provides a selective benefit, and similar stabilizing effects have been demonstrated for other chromosomal TA systems (60 – 63).

Overall, our work provides a detailed characterization of the HipBST TA system in *L. pneumophila*. Given its small and unstudied TA system set, extreme genomic characteristics, and challenging host-associated lifestyle, investigating the TA systems in this species can be a source of new insights into TA biology and function. We find a strong signal of conservation for HipBST within the *Legionella* genus, which is unusual for a TA system and, in particular, one that is almost completely absent from all other taxa in the Gammaproteobacteria. Despite its frequent occurrence in accessory genomic clusters, this level of conservation suggests that some functionality beyond promiscuous acquisition and addiction is being selected for. We also show that considerable molecular divergence can be found between related or even the same TA system family, which underscores the potential for discovering new biology in the expansive and unexplored sequence space of bacterial genomes. These findings, therefore, justify caution when generalizing TA system functionality across different bacterial hosts and instead suggest that each system be considered within the context of its specific genomic niche.

## MATERIALS AND METHODS

### Strains and plasmids

All strains used in this study are listed in Table S1. All plasmids used in this study are listed in Table S2. All oligonucleotides used in this study are listed in Table S3. *L. pneumophila* strains used were derived from Lp01^JK^ (64), except for Lp02, which was used for the TEM-1 translocation experiments. *E. coli* XL1-Blue and TOP10 cells (Invitrogen) were used for cloning and plasmid maintenance, and BL21-GOLD (DE3) (Stratagene) cells were used for protein expression. TOP10 and BL21-GOLD (DE3) were additionally used for *in vivo* toxicity assays. *Saccharomyces cerevisia*e Y8800 (*MATa leu2-3,112 trp1-901 his3-200 ura3-52 gal4∆ gal80∆ GAL2-ADE2 LYS2::GAL1-HIS3 MET2::GAL7-lacZ cyh2R*) (65) was used for yeast two-hybrid assays and BY4742 (*MATα his3Δ1 leu2Δ0 met15Δ0 ura3Δ0*) (66) was used for *in vivo* toxicity assays. Bacterial expression plasmids were constructed using PCR products amplified from Lp01^JK^ genomic DNA with restriction cloning, or ligation-independent cloning for the pMCSG68-SBP-TEV protein purification constructs. PCR products were cloned into pDONR221-ccdB using BP clonase (Invitrogen). The resulting pDONR221 constructs were then used to clone the *hipBST_Lp_* genes into the yeast expression vectors pAG425GAL-ccdB (for yeast toxicity tests), pAG416GPD-ccdB, pDEST-DB-ccdB, and pDEST-AD-ccdB (for the Y2H and Y3H assays) using LR clonase (Invitrogen). pAG425GAL-ccdB (Addgene plasmid # 14153; RRID:Addgene_14153) and pAG416GPD-ccdB (Addgene plasmid # 14148; RRID:Addgene_14148) (67) were gifts from Susan Lindquist. Vectors with the DNA-binding (DB) and transcription-activating (AD) domain of Gal4 (pDEST-DB, pDEST-AD) (68) were a kind gift from N. Yachie and F. Roth (University of Toronto, Canada). Genetic substitutions were introduced via site-directed mutagenesis using the Q5 site-directed mutagenesis kit (NEB). All generated constructs were confirmed by Sanger sequencing. Plasmids were introduced into *E. coli* via heat-shock transformation, into *S. cerevisia*e via the PEG/LiAc method (69), and into *L. pneumophila* via electroporation. The endogenous *hipBST* locus in *L. pneumophila* was deleted using a previously described genome editing method (70) with minor modifications (71). The resulting deletion mutant was screened by PCR using the oligos JL-P102 and JL-P103, and validated with Sanger sequencing using the oligo JL-P101.

### Media and culture conditions

Bacterial experiments and routine strain maintenance were performed at 37°C. *L. pneumophila* strains were grown in *N*-(2-acetamido)-2-aminoethanesulfonic acid (ACES)-buffered yeast extract and on charcoal AYE agar plates supplemented with 0.4 g/L L-cysteine and 0.25 g/L ferric pyrophosphate. For liquid growth, cultures were inoculated from patches grown for 2 days. When required for selection or plasmid maintenance, media were supplemented with chloramphenicol (5 µg/mL), kanamycin (20 µg/mL), or thymidine (100 µg/mL). Ectopic gene expression was induced by isopropylthio-β-galactoside (IPTG; 100 µM) and repressed with glucose (1% vol/vol), unless otherwise indicated. *E. coli* strains were grown on lysogeny broth (LB, Miller) liquid media and agar. When required, media were supplemented with ampicillin (100 µg/mL), chloramphenicol (34 µg/mL), or kanamycin (40 µg/mL) for selection or plasmid maintenance. Ectopic gene expression was induced by arabinose (0.2% wt/vol) or IPTG (100 µM) and repressed with 1% glucose unless otherwise indicated. Yeast experiments and routine strain maintenance were performed at 30°C. *S. cerevisia*e strains were grown on yeast peptone adenine dextrose medium (2% bacto peptone wt/vol, 1% yeast extract wt/vol, 2% glucose vol/vol, 180 mg/L adenine sulfate), or synthetic defined (SD) medium composed of yeast nitrogen base with ammonium sulfate, supplemented with 2% glucose and all amino acids, lacking specific amino acids where necessary for selection or plasmid maintenance. When required, media lacking glucose were supplemented with galactose (2% vol/vol) to induce gene expression.

### TEM-1 β-lactamase translocation assays

Protein translocation was tested as described previously (39). Briefly, Lp02 strains carrying pXDC61 constructs containing the TEM-1 β-lactamase fused to a gene of interest were grown overnight to mid-log phase (OD_600_ = 1.5–2), and fusion protein expression was induced with IPTG (500 µM) for 3 h. The cultures were then used to infect monolayers of differentiated U937 cells (RPMI 1640 medium, Gibco) in triplicate at various MOIs (20, 50, and 125) for 1 h at 37°C with 5% CO_2_. LiveBLAzer dye (ThermoFisher K1095) was subsequently added to the cells and incubated for another 2 h, followed by blue/green fluorescence detection using a TECAN Infinite M PLEX plate reader (blue fluorescence: 409 nm excitation, 460 nm emission, gain 140, integration time 20 μs; green fluorescence: 409 nm excitation, 530 nm emission, gain 140, integration time 20 μs). A blue/green fluorescence ratio >1 was used as the threshold to determine translocation (55). Each experiment was performed in triplicate and repeated three times. Immunoblotting to confirm TEM-1 fusion protein expression was performed with an anti-β lactamase antibody (Abcam 12251).

### *In vivo* bacterial toxicity experiments

Growth assays were performed as follows: *E. coli* and *L. pneumophila* strains containing plasmids expressing the genes of interest were grown overnight in the presence of 1% glucose. Cultures were washed to remove glucose and adjusted to OD_600_ = 0.1 in fresh media supplemented with either 1% glucose or arabinose/IPTG. Cultures were plated in triplicate in a flat bottom 96-well plate (100 µL volume), sealed with a Breathe-Easy sealing membrane (Diversified Biotech BEM-1), and optical density (600 nm) was monitored every 15 min for 24 h using an S&P growth curve robot. Each experiment was repeated a minimum of three times unless otherwise indicated.

### Protein expression and purification for *in vitro* experiments

Protein expression and purification were performed as described previously (72). Briefly, BL21-GOLD (DE3) cells carrying the desired His_6_-SBP-tagged constructs were grown at 37°C to OD_600_ = 0.5, at which time protein expression was induced with IPTG (1 mM) for 5 h at 37°C. Cells were harvested by centrifugation (12,227 × *g* for 10 min at 4°C), resuspended in lysis buffer {300 mM NaCl, 5% glycerol, 5 mM imidazole, 50 mM HEPES [4-(2-hydroxyethyl)−1-piperazineethanesulfonic acid] pH 7.5}, lysed by sonication on ice (30% amplitude, 10 s on, 10 s off for 5 min) in the presence of 1 mM phenylmethylsulfonyl fluoride (PMSF), and the soluble fraction was obtained by high-speed centrifugation (34,957 × *g* for 20 min at 4°C). The tagged proteins were purified by immobilized metal affinity chromatography using nickel-nitrilotriacetic acid beads. Columns were washed with wash buffer (300 mM NaCl, 30 mM imidazole, 15 mM HEPES pH 7.5, 5% glycerol), and eluted in 300 mM NaCl, 300 mM imidazole, and 15 mM HEPES pH 7.5. Eluted protein was then dialyzed in 300 mM NaCl, 15 mM HEPES pH 7.5, 0.5 mM dithiothreitol (DTT), and concentrated using Vivaspin 5 kDa (HipB_Lp_, HipS_Lp_) and 10 kDa (HipT_Lp_) cutoff columns (GE Healthcare). Protein concentration was quantified using the Bradford assay (BioShop). For additional purification (*in vitro* assays involving co-incubation), His_6_-SBP-tagged HipB_Lp_, HipS_Lp_, and HipT_Lp_ were injected into a Superdex S75 10/300 GL size exclusion column (GE Healthcare) equilibrated in dialysis buffer, and the purified fractions were collected.

### Crystallography and structure determination

The HipS_Lp_-HipT_Lp_ complex was crystallized as selenomethionine (Se-Met)-derivatized proteins and as native proteins (for the higher resolution final structure). Cloned in pETDuet vector, HipS_Lp_ and His_6_-HipT_Lp_ proteins were expressed in *E. coli* BL21(DE3)-Magic cells. For the (Se-Met)-derivatized protein complex, cells were grown in M9 minimal media (Shanghai Medicilon) with 1 mM IPTG induction at 20°C when the OD_600_ reached 1.2. The native protein complex was expressed in ZYP-5052 auto-inducing complex medium (73) by incubating for a few hours at 37°C followed by overnight growth at 20°C. Overnight cell culture was then collected by centrifugation at 6,000 × *g* for 25 min at 4°C. Cells were resuspended in a binding buffer [100  mM HEPES (pH 7.5), 500  mM NaCl, 5  mM imidazole, and 5% glycerol (vol/vol)]. The purification was performed as described above, which resulted in the formation of the HipS_Lp_-HipT_Lp_ complex as judged by SDS-PAGE. HipB_Lp_ was expressed as a native protein and purified as described above. Crystals were grown at room temperature (RT) using the vapor diffusion sitting drop method. For the HipS_Lp_-HipT_Lp_ (Se-Met) complex, 17 mg/mL protein was mixed with reservoir solution [0.1 M potassium chloride, 10 mM Tris pH 7, 20% (vol/vol) PEG 4K]. For the HipS_Lp_-HipT_Lp_ (native) complex, 8 mg/mL protein was mixed with reservoir solution [0.1 M HEPES pH 7.5 and 30% (vol/vol) PEG 1K]. For the HipB_Lp_ crystal, 20 mg/mL protein was mixed with reservoir solution [0.2 M ammonium sulfate, 0.1 M sodium acetate pH 4.6, and 25% (vol/vol) PEG 4K]. Crystals were cryoprotected with paratone oil. For the HipS_Lp_-HipT_Lp_ complex crystal, diffraction data at 100 K were collected at beamline 19-ID of the Structural Biology Center at the Advanced Photon Source, Argonne National Laboratory. For the HipB_Lp_ crystal, diffraction data at 100 K were collected at a home source Rigaku HF-007 rotating anode with Rigaku R-AXIS IV image plate detector. All diffraction data were processed using HKL3000 (74). The HipS_Lp_-HipT_Lp_ (Se-Met) complex was solved using Phenix.Autosol (75), which identified seven Se-Met sites out of seven total methionine residues in the asymmetric unit; the native complex was solved by molecular replacement using the Se-Met complex structure. The structure of HipB_Lp_ was solved by molecular replacement using the CCP4 online Balbes server and the structure of HipB_Ec_ (PDB 2WIU). All model building and refinement were performed using Phenix.refine and Coot (76).

### *In vitro* kinase assays

Purified recombinant His_6_-SBP-tagged HipB_Lp_, HipS_Lp_, and HipT_Lp_ (WT, D197Q, D219Q) were tested for ATP hydrolytic activity using the ADP-Glo kinase assay (Promega). Briefly, 1 µg of each protein was assayed in 25 µL reactions containing 40 mM Tris-HCl pH 7.5, 20 mM MgCl_2_, 0.1 mg/mL bovine serum albumin, 1 mM DTT, and 25 µM ATP. When included, the kinase inhibitor FSBA was supplemented to a final concentration of 20 mM. Reactions were incubated at 37°C for 30 min and luminescence was measured using a TECAN Infinite M PLEX plate reader (500 ms integration time). Each experiment was replicated independently a minimum of two times.

### Electrophoretic mobility shift assays

Varying concentrations of purified recombinant His_6_-SBP tag-cleaved HipB_Lp_, HipS_Lp_, and HipT_Lp_ were incubated with double-stranded DNA substrates (gel purified PCR products or annealed oligos) in binding buffer (400 mM KCl, 150 mM HEPES pH 7.5, 10 mM EDTA, 50% glycerol, 5 mM DTT) for 30 min at RT. The protein-DNA mixtures were then run at 70 V on a 6% native polyacrylamide gel with 1× TAE (Tris-acetate-EDTA) running buffer, stained with SYBR Green (Invitrogen), and imaged. DNA substrates consisted of the promoter region of the *hipBST_Lp_* operon (upstream 200 bp amplified with oligos JL-P336 and JL-P341) or two other predicted toxin-antitoxin operons used as controls (*lpg1604-05* and *lpg1934-35* upstream 200 bp amplified with oligos JL-P516/JL-P517 and JL-P518/JL-P519, respectively). The 50-bp fragments containing DNA sequence from upstream of the *hipBST_Lp_* operon were generated by annealing complementary oligos (Table S3) in a 1:1 ratio in annealing buffer (100 mM KAc, 30 mM HEPES pH 7.5), heating at 94°C for 2 min, and cooling slowly to RT. Each experiment was repeated a minimum of three times.

### Yeast two-hybrid assays

Yeast two-hybrid experiments were performed as described previously (77). Briefly, proteins of interest were fused to either the GAL4 transcriptional AD or DB domain, using the pDEST-AD-ccdB and pDEST-DB-ccdB constitutively active Gateway destination plasmids. Three independent clones of Y8800 containing pDEST-AD and pDEST-DB encoding gene fusions of interest were grown overnight at 30°C in liquid SD-Leu/Trp media supplemented with 2% glucose. These cultures were then stamped on plates containing (control) or lacking histidine (physical interaction selection). When required, a third protein was expressed constitutively from the pAG416GPD plasmid. Y8800 strains containing pDEST-AD, pDEST-DB, and pAG416GPD together were grown with SD-Leu/Trp/Ura media supplemented with 2% glucose. Plates were imaged after 2 days of growth at 30°C. Each experiment was repeated a minimum of two times.

### Yeast spotting assays

Yeast spotting experiments were performed as described previously (77). Briefly, three independent clones of BY4742 containing the gene of interest in the pAG425GAL expression vector were grown in triplicate overnight at 30°C in liquid SD-Leu media supplemented with 2% glucose and adjusted to OD_600_ = 1 the following day. Fivefold serial dilutions of each culture were then prepared in a 96-well plate at a volume of 120 µL and stamped onto solid SD-Leu media supplemented with either glucose (2%; suppression) or galactose (2%; expression) using a VP 407AH pin tool (VP Scientific). Plates were imaged after 2 days of growth at 30°C. Each experiment was repeated a minimum of three times.

### ASKA genomic library rescue screen

The *E. coli* ASKA library (without GFP tags) (78) was pooled to a final concentration of 100 ng/µL, and 150 ng of the pool was electroporated into BL21-GOLD (DE3) cells containing HipT_Lp_ cloned into the pCDF1-b expression vector. Cells were recovered for 1 h at 37°C and transformations were plated on solid media containing either IPTG (100 µM) for gene expression or 1% glucose for repression and quantifying transformation efficiency. As a control, the empty vector pCA24N was transformed in an equivalent manner to the pooled library. Library transformations without gene expression yielded 10^4^–10^5^ cells and library transformations were performed a minimum of three times for each experiment. Individual screening experiments were repeated three times. Transformants that grew under selective conditions were re-struck on selective media to ensure a stable rescue phenotype. Plasmids from the resulting strains were then prepared and Sanger sequenced using the oligo JL-P206.

### HipT_Lp_ *in vivo* expression for phosphoproteomic analysis

Cultures of *L. pneumophila* ∆*hipBST* carrying either pJB1806 or pJB1806::*hipT*_*Lp*_ were grown in the presence of 0.5% glucose until mid-log phase (OD_600_ = 1.5–2). Expression was then induced with IPTG (100 µM) and 75 mL of cells was harvested prior to and 75 min postinduction. Lysates were prepared by resuspending cell pellets in 2 mL NP-40 buffer [50 mM Tris-HCl pH 7.8, 150 mM NaCl, 1% NP-40, 1 mM PMSF, 1× PhosSTOP (Roche)], sonication on ice (5 s on/10 s off for 2 min at 40% amplitude), and high-speed centrifugation (13,000 × *g* for 30 min at 4°C). Each experiment was repeated two times.

### In-solution protein digestion

Cell lysates from the ∆*hipBST* cultures (vector-induced, HipT_Lp_-uninduced, and HipT_Lp_-induced) were quantified for total protein (Pierce BCA Protein Assay Kit, Thermo Scientific), and 2.5 mg of total protein from each sample was used. Samples were reduced with 5 mM DTT (37°C, 1 h) and alkylated with 10 mM iodoacetamide (25°C, 45 min). Samples were digested for 2 h at 25°C with constant agitation with a Trypsin/LysC mix (V5071, Promega) and overnight at 25°C with proteomics-grade porcine trypsin (T6567, Sigma-Aldrich). For both digestions, 6.25 µg of trypsin was used. Samples were desalted with 50-mg capacity Sep-Pak C18 cartridges (Waters Corporation). In brief, samples were acidified with trifluoroacetic acid (TFA) to a final concentration of 1% and centrifuged at 21,000 × *g* to remove precipitate. Columns were conditioned by passing one column volume (CV) of 100% acetonitrile (ACN), one CV of 50% ACN/0.1% formic acid (FA), and four CV of 0.1% TFA. Samples were loaded and desalted with 1% TFA and 1% FA, and washed peptides were eluted with 50% ACN/0.1% FA and concentrated by vacuum centrifugation.

### Dimethyl labelling

Samples were labeled as described previously (79) with some modifications. Peptides were resuspended in 50 mM HEPES pH 8.0, and the HipT_Lp_-uninduced, vector-induced, and HipT_Lp_-induced samples were designated to be labeled with the light, intermediate, and heavy channels, respectively. Peptides were mixed with freshly prepared CH_2_O (light, +28.0313 Da), CD_2_O (intermediate, +32.0564 Da), and ^13^CD_2_O (heavy, +36.0757 Da), and all solutions were made to 4% (vol/vol). NaBH_3_CN (0.6 M) was added to the light and intermediate channels, and NaBD_3_CN (0.6 M) was added to the heavy channel. Samples were labeled at RT for 2 h, quenched with FA (5% final), and desalted with Sep-Pak C18 Cartridges. Labeled peptides were quantified (Pierce Colorimetric Peptide Assay Kit, Thermo Scientific) and mixed 1:1:1 based on peptide amount.

### Phosphopeptide enrichment

Labeled and mixed peptides were phosphoenriched with TiO_2_ magnetic beads according to the manufacturer’s protocols (MagReSyn). In brief, beads (100 µL) were equilibrated by three washes with loading buffer (0.1 M lactic acid in 80% ACN/5% TFA) before beads were incubated with 480 µg of peptides for 20 min at RT with end-over-end mixing. Samples were washed with 100 µL loading buffer, 80% ACN/1% TFA, and 10% ACN/0.2% TFA. Phosphopeptides were eluted twice in 1% NH_4_OH and acidified with FA to a final concentration of 2.5%. Samples were vacuum centrifuged and desalted with C18 stagetips. Stagetips were activated with 100% ACN/0.1% FA (buffer B) and washed twice with 5% FA. Samples resuspended in 5% FA were passed through each tip and washed with 0.1% FA (buffer A). Samples were eluted with a 2:1 mixture of buffer B and buffer A, and the eluate was vacuum centrifuged until dry.

### LC-MS/MS

For data-dependent acquisition (DDA) liquid chromatography-tandem mass spectrometry (LC-MS/MS), labeled, phoshoenriched peptides were analyzed using a nano-HPLC coupled to MS. Coated nano-spray emitters were generated from fused silica capillary tubing (75 µm ID, 365 µm OD) with a 5–8 µm tip opening, using a laser puller (Sutter Instrument Co., model P-2000). Nano-spray emitters were packed with C18 reversed-phase material (Reprosil-Pur 120 C18-AQ, 3 µm) and resuspended in methanol using a pressure injection cell. The sample in 5% FA was directly loaded at 400 nL/min for 20 min onto a 75 µm × 15 cm nano-spray emitter. Peptides were eluted from the column with an ACN gradient generated by an Eksigent ekspert nanoLC 425 and analyzed on an Orbitrap Fusion Lumos Tribrid mass spectrometer (ThermoFisher). The gradient was delivered at 200 nL/min from 2.5% ACN with 0.1% FA to 35% ACN with 0.1% FA using a linear gradient of 120 min. This was followed by an 8-min gradient from 35% ACN with 0.1% FA to 80% ACN with 0.1% FA. Afterward, there was an 8-min wash with 80% ACN with 0.1% FA and equilibration for another 23 min to 2.5% ACN with 0.1% FA. The total DDA protocol was 180 min. The MS1 scan had an accumulation time of 50 ms within a mass range of 400–1500 Da, using an orbitrap resolution of 120,000, 60% RF lens, AGC target of 125%, and 2400 volts. This was followed by MS/MS scans with a total cycle time of 3 s. Accumulation time of 50 ms and 33% HCD collision energy were used for each MS/MS scan. Each candidate ion was required to have a charge state from 2-7 and an AGC target of 400%, isolated using an orbitrap resolution of 15,000. Previously analyzed candidate ions were dynamically excluded for 9 s.

### MS data processing and analysis

Data files were processed and searched using MaxQuant (version 2.1.4.0) querying against *Legionella pneumophila* sequences (RefSeq accession: GCF_001941585.1) that were modified to include common contaminants and reverse sequences (FragPipe v.18.0). Search parameters were set to search for tryptic cleavages allowing two missed cleavages. Variable modifications included methionine oxidation, protein N-terminal acetylation, asparagine deamidation, serine, threonine, and tyrosine phosphorylation, and lysine dimethylation. Fixed modifications included carbamidomethylation. A maximum of five modifications per peptide was allowed. Other settings were left as default parameters. For dimethyl labeling, light labels were processed with DimethLys0/Nter0, intermediate labels with DimethLys4/Nter4, and heavy labels with DimethLys8/Nter8. Biological duplicates were processed separately by assigning different parameter groups with posttranslational modifications (PTM) set to TRUE. Peptides that were flagged as contaminants, not labeled as phosphorylated or only found in one biological replicate were filtered out, and the intensity columns (e.g., Intensity.H) were used for analysis. Data analysis and plotting were conducted in R (version 4.2.0).

### HipBST conservation and accessory genome search across *Legionella* species

Genome assemblies for 58 representative *Legionella* species were retrieved in July 2020 from the NCBI RefSeq database (Table S4). When possible, genome assemblies with higher sequencing coverage (23) were used instead. For *Legionella pneumophila*, a recently reannotated genome (Refseq ID: GCF_001941585.1) was used. ORFs were predicted using Prodigal (80) and homologs were determined using OrthoMCL (25). A core genome phylogeny was constructed by aligning all conserved ORFs with MUSCLE and building a phylogenetic tree with RAxML (JTTDCMUT model selected by PROTGAMMAAUTO) (27), consistent with previous work (22). The resulting tree was annotated using the R package ggtree (28). All software was run with default parameters. To identify non-essential gene clusters, genes in *L. pneumophila* were first annotated as essential if they were reported to demonstrate a growth defect in broth (50, 51) or replication within a host (51) when deleted or were conserved across all 58 species. These annotations were then applied to homologous groups across all *Legionella* genomes. To predict non-essential clusters, a 10-gene sliding window analysis was performed on each genome and used to define regions where the local maximum of essential genes never exceeded 10%. In addition, each region was required to be larger than 1% of the total genome size. Gene neighborhood analysis across *Legionella* species with HipBST systems was performed using FlaGs (29) with default parameters to retrieve 10 genes upstream and downstream of *hipS* in each genome.

### Phylogenetic analysis of HipBST systems across bacterial species

The NCBI RefSeq database was queried using cblaster (default parameters) (30) in November 2021 to retrieve homologous sequences of HipBA or HipBST systems. We excluded systems that have an intergenic distance of above 150 bp and/or contained partial protein sequences. Five seed templates were used for this search: *E. coli* K12 HipBA (HipBA_Ec_; NP_416025.1, NP_416024.1), *Shewanella oneidensis* MR-1 HipBA (HipBA_So_; AAN53783.2, AAN53784.1), *Bacteroides uniformis* HipBA (HipBA_Bu_; WP_149924066.1, WP_149924064.1), *E. coli* O127 HipBST (HipBST_Ec_; WP_000563102.1, WP_001346664.1, WP_001262465.1), and *L. pneumophila* HipBST (HipBST_Lp_; AAU28429, AAU28430, AAU28431). We first confirmed that no hit accession or sequence was retrieved by multiple system seeds in order to ensure that no ambiguity in hit assignment occurred and that each retrieved hit corresponded to only one seed. The resulting protein sequences were clustered using MMseqs2 (31) to identify sequence-level representatives (80% identity, 80% coverage, coverage mode = 1), aligned with MAFFT (E-INS-i) (32), and trimmed with trimAl (automated1 for parameter selection) (33). HipB sequences were aligned separately and the HipS/HipT sequences were concatenated prior to alignment with the HipA sequences. A HipBA/HipBST phylogeny was inferred via the maximum likelihood method using IQ-TREE (34). We used the edge-proportional partition model and performed partitioned phylogenetic analysis by segregating the protein sequence alignments into three regions: the HipB alignments, the HipA N-terminus/HipS alignments, and the HipA C-terminus/HipT alignments. Branch confidence was assessed using an ultrafast bootstrap with 1,000 replications. The final tree was then manually inspected to ensure that all representative hits clustered with their respective seeds and that there was no ambiguity in the assignment of a hit to a given seed system. As a check for congruence, individual phylogenies were constructed for HipB and HipA/HipST (concatenated) homologs using the same protocol for alignment and trimming (see above). Individual maximum likelihood phylogenetic trees for HipB and HipA/HipST were constructed as above and ModelFinder (81) was used to determine the best partitioning scheme. To explore the phylogenetic distribution of bacteria harboring each system, species-level representatives of every taxon containing a system homolog from the initial search results were used to retrieve a phylogenetic tree from TimeTree (36). A second tree was retrieved after filtering for species with complete genome assemblies according to the NCBI Microbial Genome database, to ensure the absence of HipBA/HipBST systems is not due to genome incompleteness. Presence or absence was then compared for each system type across species and represented using the R package ggtree (28).

## Data Availability

All data have been deposited as a complete submission to the MassIVE repository (https://massive.ucsd.edu/ProteoSAFe/static/massive.jsp) and assigned the accession number MSV000090735. Atomic coordinates have been deposited in the Protein Data Bank with accession codes 8EZR, 8EZS, and 8EZT.

## SUPPLEMENTAL MATERIAL

Supplemental figures mbio.01510-23-s0001.pdf Figures S1-S8.

Table S1 mbio.01510-23-s0002.xlsx Strains used in this study.

Table S2 mbio.01510-23-s0003.xlsx Plasmids used in this study.

Table S3 mbio.01510-23-s0004.xlsx Oligonucleotides used in this study.

Table S4 mbio.01510-23-s0005.xlsx Legionella genome assemblies.

Table S5 mbio.01510-23-s0006.xlsb HipBA and HipBST homology search results.

Table S6 mbio.01510-23-s0007.xlsx Phylogeny search results for HipBA and HipBST containing species.

Table S7 mbio.01510-23-s0008.xlsx X-ray diffraction data.

Table S8 mbio.01510-23-s0009.xlsx Phosphoproteomic screen results.

ASM does not own the copyrights to Supplemental Material that may be linked to, or accessed through, an article. The authors have granted ASM a non-exclusive, world-wide license to publish the Supplemental Material files. Please contact the corresponding author directly for reuse.
