# Supplementary material for: Functional diversification despite structural congruence in the HipBST toxin-antitoxin system of Legionella pneumophila
Source: mBio. 2023 Oct 11;14(5):e01510-23. doi: 10.1128/mbio.01510-23 (PMC10653801; doi:10.1128/mbio.01510-23)
Supplement: Supplemental figures — Figures S1-S8. [file mbio.01510-23-s0001.pdf]

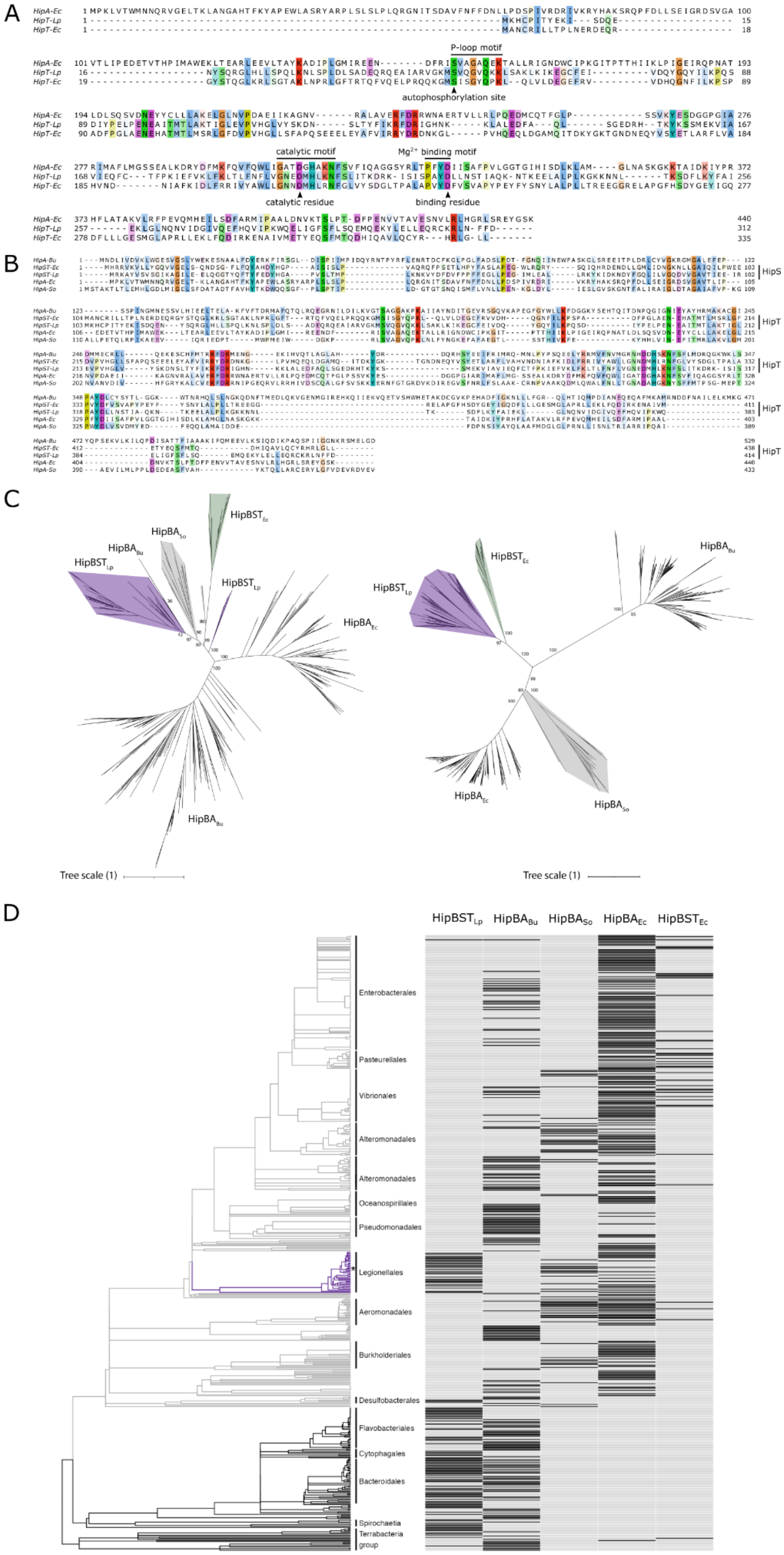
A
HipA-Ec
HipT-Lp
HipT-Ec
P-loop motif
autophosphorylation site
catalytic motif
Mg2+ binding motif
catalytic residue
binding residue
B
HipA-Bu
HipST-Ec
HipST-Lp
HipA-Ec
HipA-So
HipS
HipT
C
HipBST_Ec
HipBA_So
HipBA_Bu
HipBST_Lp
HipBA_Ec
Tree scale (1)
D
HipBST_Lp
HipBA_Bu
HipBA_So
HipBA_Ec
HipBST_Ec
Enterobacterales
Pasteurellales
Vibrionales
Alteromonadales
Alteromonadales
Oceanospirillales
Pseudomonadales
Legionellales
Aeromonadales
Burkholderiales
Desulfobacterales
Flavobacteriales
Cytophagales
Bacteroidales
Spirochaetia
Terrabacteria group

Figure S1. Conservation and divergence among the Hip toxins.

(A) Alignments of $HipA_{Ec}$ (NP_416024.1), $HipT_{Lp}$ (AAU28431), and $HipT_{Ec}$ (WP_001262465.1) constructed with MUSCLE. Residues are coloured with the Clustal X colour scheme implemented in Jalview (>33% identity) (1). Conserved residues and motifs are indicated. **(B)** Comparison of the seed sequences used to search for HipBA and HipBST homologs. Alignments of $HipA_{Bu}$ (WP_149924064.1), $HipA_{Ec}$ (NP_416024.1), $HipA_{So}$ (AAN53784.1), $HipST_{Ec}$ (WP_001346664.1, WP_001262465.1), and $HipST_{Lp}$ (AAU28430, AAU28431) constructed with MUSCLE. Residues are coloured with the Clustal X colour scheme implemented in Jalview (>40% identity). **(C)** Unrooted phylogenies of HipB (left) and HipA/HipST (right) homolog sequences used to construct the phylogeny in Figure 1C. HipS and HipT sequences were concatenated prior to alignment with HipA sequences. The phylogenies were constructed using IQ-TREE and visualized with iTOL. The scale bar denotes substitutions per site. Bootstrap values are indicated for major nodes and the systems from which each homolog clade is derived are noted. **(D)** Distribution of HipBA and HipBST TA systems across diverse bacterial taxa (as in Figure 1D), but without any filtering of species based on genome completeness. The bacterial phylogeny was retrieved from TimeTree for all species containing at least one system in our homology search and the presence of each system homolog is indicated for each species (data available in Table S6). Systems are ordered by similarity of taxonomic distribution. The Pseudomonadota phylum is coloured light grey, the Legionellales order is coloured purple, and *L. pneumophila* is indicated with an asterisk.

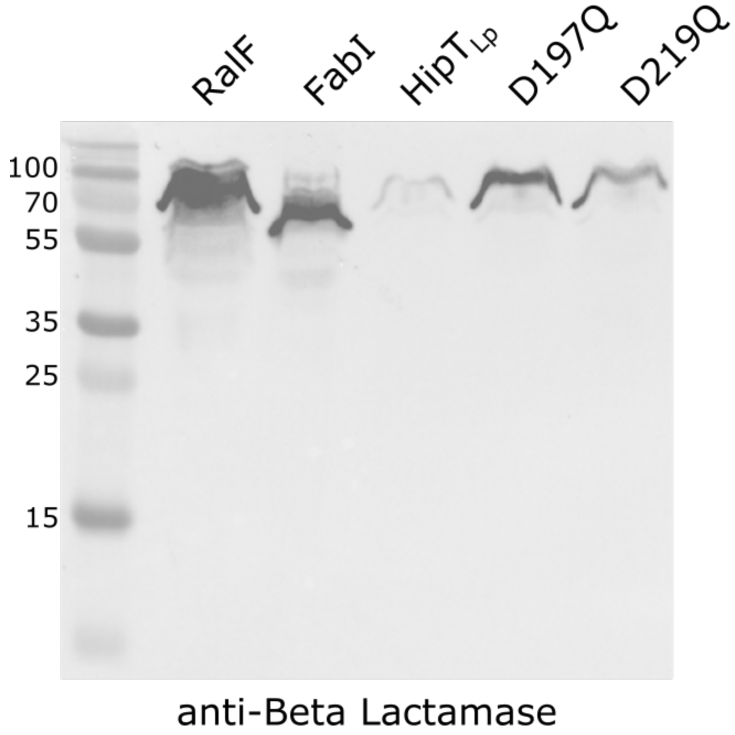

Figure S2. TEM-1 fusion proteins are expressed in *L. pneumophila* cells prior to translocation assays. Western blot of TEM-1 fusion protein expression in *L. pneumophila* Lp02 cells using an anti-Beta Lactamase antibody (Abcam 12251). Fusion protein expression from the pXDC61 vector was induced with IPTG (500 µM) for 3 hr. Just prior to infection of U937 monolayers, samples of induced cells were harvested for immunoblotting. The predicted molecular weight of the TEM-1 β-lactamase is approximately 31.5 kDa.

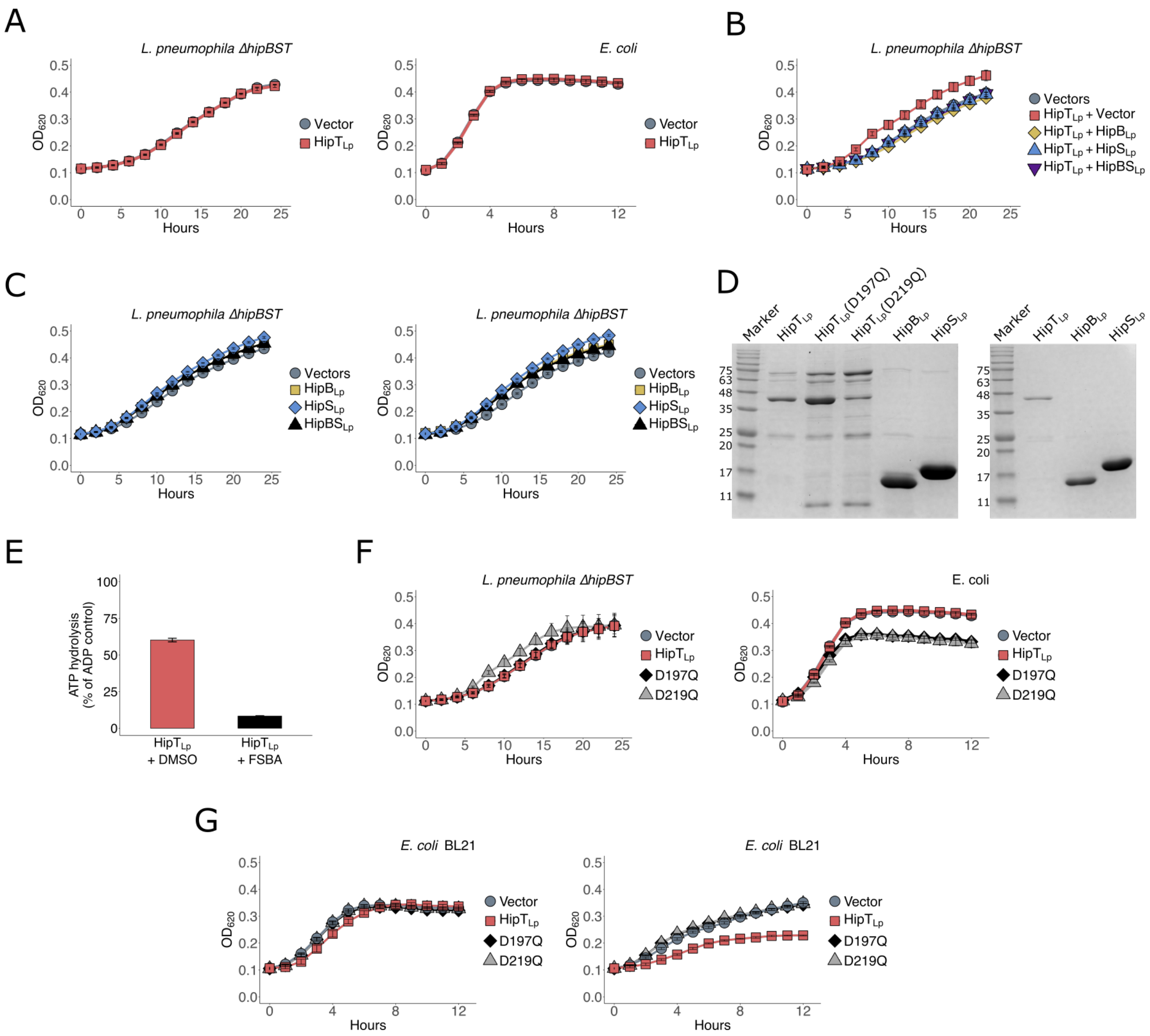
A
L. pneumophila ΔhipBST
E. coli
OD620
Hours
Vector
HipTLp
B
L. pneumophila ΔhipBST
Vectors
HipTLp + Vector
HipTLp + HipBLp
HipTLp + HipSLp
HipTLp + HipBSLp
C
L. pneumophila ΔhipBST
Vectors
HipBLp
HipSLp
HipBSLp
D
Marker
HipTLp
HipTLp(D197Q)
HipTLp(D219Q)
HipBLp
HipSLp
75
63
48
35
25
20
17
11
E
ATP hydrolysis (% of ADP control)
HipTLp + DMSO
HipTLp + FSBA
F
L. pneumophila ΔhipBST
E. coli
Vector
HipTLp
D197Q
D219Q
G
E. coli BL21

Figure S3. HipBST$_{Lp}$ is a functional tripartite toxin-antitoxin system.

(A) Uninduced controls of HipT$_{Lp}$ expression vectors in *L. pneumophila* Δ*hipBST* (pJB1806) and *E. coli* TOP10 (pBAD18) cells. Expression was repressed with 1% glucose for all constructs. **(B)** Uninduced controls of strains co-expressing HipT$_{Lp}$ with HipS$_{Lp}$ and HipB$_{Lp}$ in *L. pneumophila* Δ*hipBST*. Expression was repressed with 1% glucose for all constructs. **(C)** Expression of HipB$_{Lp}$, HipS$_{Lp}$, and HipBS$_{Lp}$ from pNT562 in *L. pneumophila* Δ*hipBST* cells. Uninduced controls repressed with 1% glucose (left) and cultures induced with 100 μM IPTG (right) are shown. All strains also carried the pJB1806 empty vector. **(D)** N-terminal His$_6$-SBP-tagged HipB$_{Lp}$, HipS$_{Lp}$, and HipBST$_{Lp}$ (wild-type and mutants) purified with nickel affinity chromatography (left gel). Proteins after secondary purification with size exclusion chromatography (right gel). Purified proteins were analyzed by SDS-PAGE and stained with Coomassie dye. **(E)** ADP-Glo kinase assay performed with purified His$_6$-SBP-tagged HipT$_{Lp}$ and the kinase inhibitor FSBA or a DMSO control. The reactions were incubated at 37°C for 30 min. Data shown are the mean ± standard deviation of 2 technical replicates and are representative of 2 independent experiments. **(F)** Uninduced controls of two HipT$_{Lp}$ catalytic mutants (D197Q, D219Q) in both *L. pneumophila* Δ*hipBST* and *E. coli* (TOP10) cells. Expression was repressed with 1% glucose for all constructs. **(G)** Expression of wild-type HipT$_{Lp}$ and two catalytic mutants (D197Q, D219Q) from the pJB1806 vector in BL21 cells. Uninduced controls repressed with 1% glucose (left) and cultures induced with 100 μM IPTG (right) are shown. All growth curves show the mean ± the standard deviation of 3 biological replicates. Data are representative of 3 independent experiments.

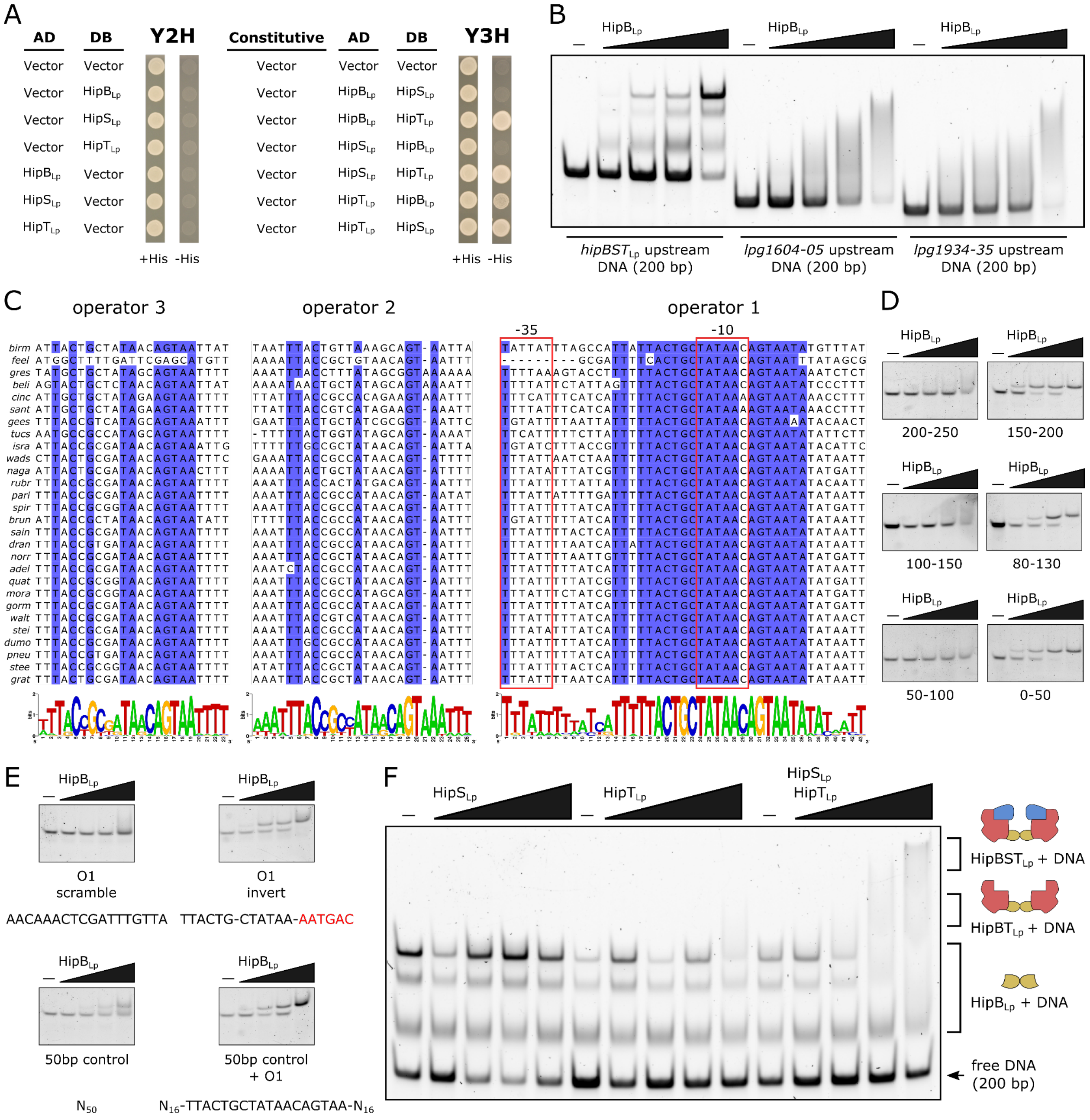
A
AD
DB
Y2H
Vector
HipBLp
HipSLp
HipTLp
+His
-His
Constitutive
Y3H
B
HipBLp
hipBSTLp upstream DNA (200 bp)
lpg1604-05 upstream DNA (200 bp)
lpg1934-35 upstream DNA (200 bp)
C
operator 3
operator 2
operator 1
-35
-10
D
200-250
150-200
100-150
80-130
50-100
0-50
E
O1 scramble
AACAAACTCGATTTGTTA
O1 invert
TTACTG-CTATAA-AATGAC
50bp control
N50
50bp control + O1
N16-TTACTGCTATAACAGTAA-N16
F
HipSLp
HipTLp
HipBSTLp + DNA
HipBTLp + DNA
HipBLp + DNA
free DNA (200 bp)

Figure S4. The HipBST$_{Lp}$ system has the capacity for complex binary and ternary regulatory dynamics. **(A)** Empty vector controls for yeast two-hybrid (Y2H) experiments testing for binary physical interactions in the HipBST$_{Lp}$ tripartite proteins. Genes cloned into the pDEST-AD and pDEST-DB Y2H vectors are indicated, and representative images are shown of *S. cerevisia*e Y8800 growth in the presence (+His) and absence (-His) of histidine. Yeast two-hybrid experiments were also performed with a third protein constitutively expressed from the pAG416 vector (Y3H). The empty pAG416 vector was used for these experiments as a control. **(B)** EMSA performed with recombinant purified HipB$_{Lp}$ and 200 bp DNA fragments (10 nM) encompassing the upstream promoter region of either the *hipBST$_{Lp}$* locus or two unrelated loci that are predicted TA systems. HipB$_{Lp}$ was added to DNA at concentrations of 1, 5, 10, and 20 nM. **(C)** Nucleotide sequence alignment of the operator sites upstream of *hipBST* systems across 28 *Legionella* species and consensus logos for each operator. Alignments were constructed with MUSCLE and residues were coloured by conservation (>95% identity) in Jalview. Bioinformatically predicted (BPROM) (2) promoter elements (-10 and -35) are indicated. **(D)** Representative EMSAs performed with 50 bp fragments from the 250 bp region upstream of *hipBST$_{Lp}$*. HipB$_{Lp}$ was added to DNA (10 nM) at concentrations of 1, 5, 10, and 20 nM. Nucleotide positions upstream of the HipB$_{Lp}$ start codon are indicated. **(E)** Representative EMSAs with 50 bp control DNA (10 nM) incubated with HipB$_{Lp}$ at concentrations of 1, 5, 10, and 20 nM. Controls consisted of the 50 bp fragment containing O1 with a scrambled O1 sequence (OS) or inversion of the downstream inverted repeat (OI; coloured red), or a 50 bp random control DNA fragment (C) with the O1 sequence added (CO). The O1 sequence, where applicable, is displayed. **(F)** Representative EMSA showing HipS$_{Lp}$ and HipT$_{Lp}$ added to HipB$_{Lp}$-DNA complexes (10 nM HipB$_{Lp}$, 10 nM DNA). HipS$_{Lp}$ and HipT$_{Lp}$ were added at concentrations of 10, 20, 40, and 80 nM. All EMSA gels were stained with SYBR Green and protein-DNA complexes are indicated. Dashes indicate the absence of added protein.

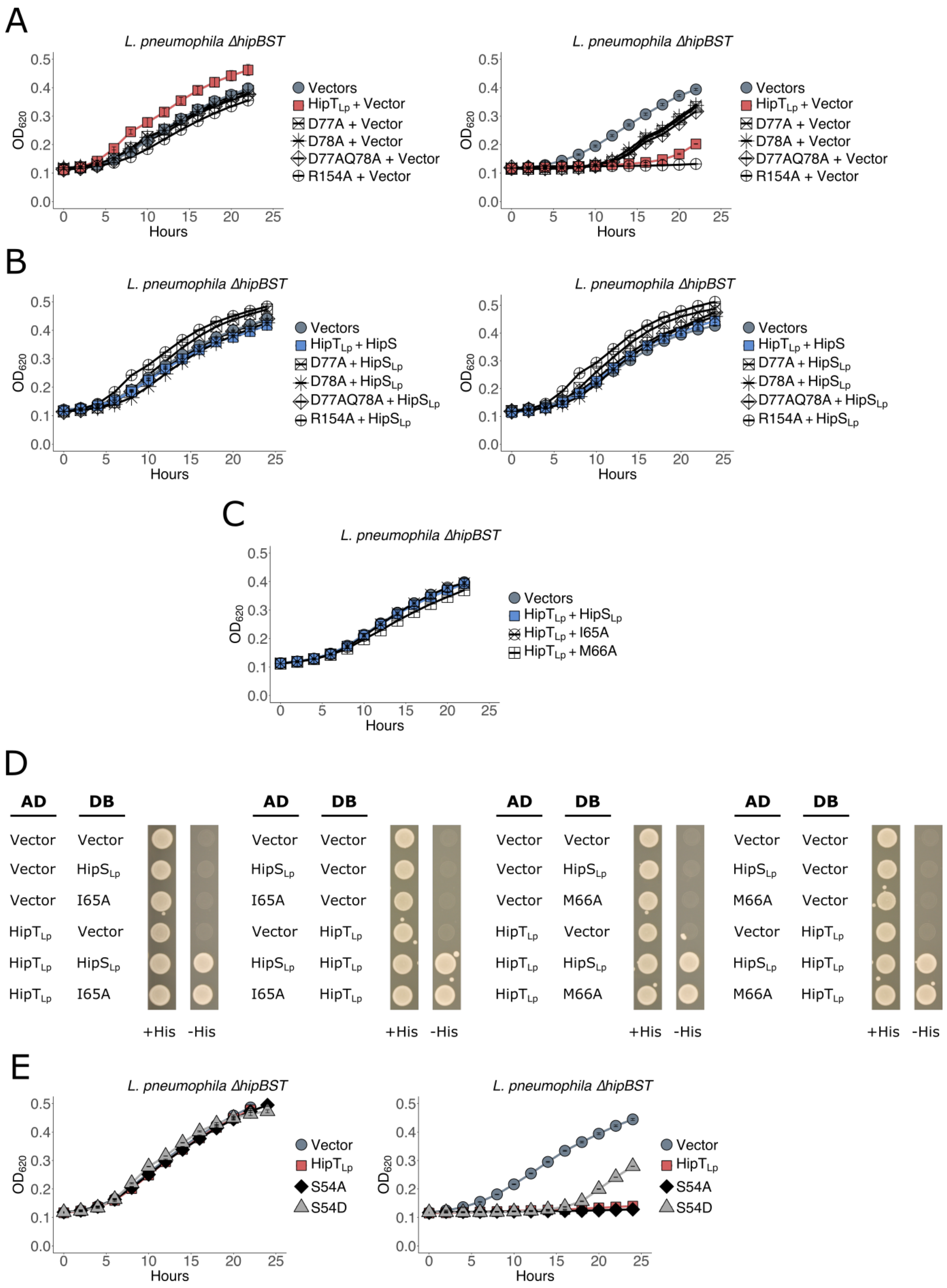

A
L. pneumophila ΔhipBST
OD620
Hours
Vectors
HipTLp + Vector
D77A + Vector
D78A + Vector
D77AQ78A + Vector
R154A + Vector
B
Vectors
HipTLp + HipS
D77A + HipSLp
D78A + HipSLp
D77AQ78A + HipSLp
R154A + HipSLp
C
HipTLp + HipSLp
HipTLp + I65A
HipTLp + M66A
D
AD
DB
Vector
HipSLp
I65A
M66A
HipTLp
+His
-His
E
Vector
HipTLp
S54A
S54D

Figure S5. Analysis of key residues involved in $HipT_{Lp}$ neutralization by $HipS_{Lp}$ and autophosphorylation. **(A)** Growth assays of *L. pneumophila* Δ*hipBST* expressing $HipT_{Lp}$ (pJB1806) with mutations in residues predicted to be important to the $HipS_{Lp}$-$HipT_{Lp}$ interaction interface. Experiments were performed to test whether these mutations affected $HipT_{Lp}$ activity. Uninduced controls (left) were repressed with 1% glucose and cultures were induced with 100 µM IPTG (right). All strains also carried the pNT562 empty vector. **(B)** Growth assays of *L. pneumophila* Δ*hipBST* expressing $HipT_{Lp}$ (pJB1806) with mutations in residues predicted to be important to the $HipS_{Lp}$-$HipT_{Lp}$ interaction interface. Mutants were co-expressed with $HipS_{Lp}$ (pNT562). Uninduced controls (left) were repressed with 1% glucose and cultures were induced with 100 µM IPTG (right). **(C)** Uninduced controls (repressed with 1% glucose) for *L. pneumophila* Δ*hipBST* co-expressing $HipT_{Lp}$ (pJB1806) with $HipS_{Lp}$ (pNT562) bearing mutations to two residues (I65A, M66A) predicted to be important to the $HipS_{Lp}$-$HipT_{Lp}$ interaction interface. **(D)** Yeast two-hybrid experiments testing for binary physical interactions between $HipT_{Lp}$ and $HipS_{Lp}$. Both wild-type $HipS_{Lp}$ and $HipS_{Lp}$ bearing mutations to two residues (I65A, M66A) predicted to be important to the $HipS_{Lp}$-$HipT_{Lp}$ interaction interface were tested. Genes cloned into the pDEST-AD and pDEST-DB Y2H vectors are indicated, and representative images are shown of *S. cerevisia*e Y8800 growth in the presence (+His) and absence (-His) of histidine. Experiments were performed in duplicate. **(D)** Growth assays of *L. pneumophila* Δ*hipBST* expressing $HipT_{Lp}$ (pJB1806) with phosphomimetic (S54D) and phosphoablative (S54A) mutations in its P-loop serine (S54). Uninduced controls (left) were repressed with 1% glucose and cultures were induced with 100 µM IPTG (right). All growth curves show the mean ± the standard deviation of 3 biological replicates. Data are representative of 3 independent experiments.

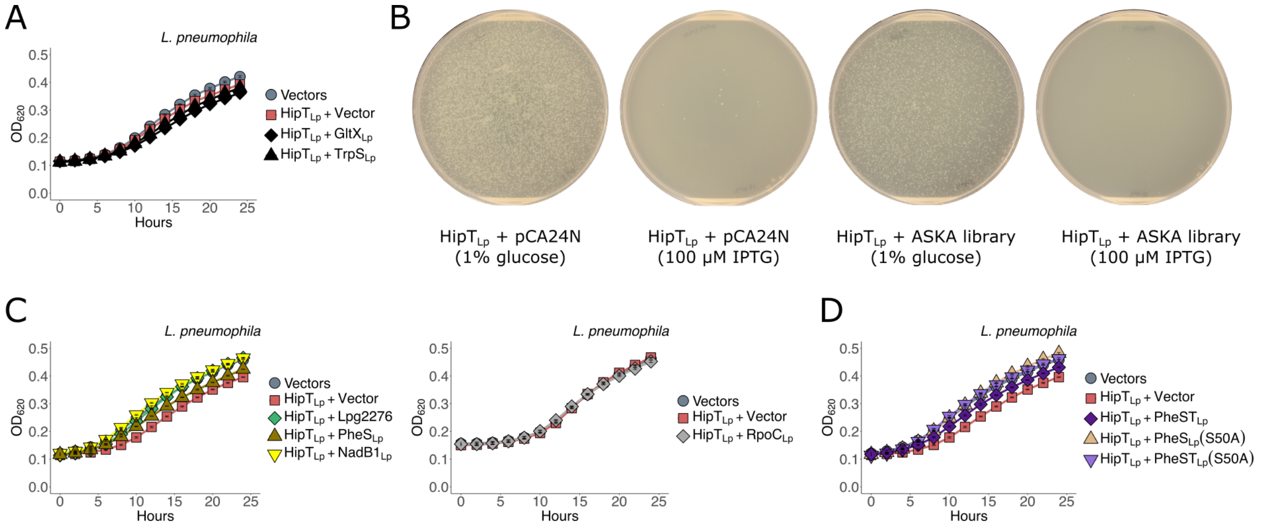

Figure S6. $HipT_{Lp}$ does not target a previously characterized substrate of HipT or HipA toxins.

(A) Uninduced controls of growth assays of *L. pneumophila* co-expressing $HipT_{Lp}$ (pJB1806) with $GltX_{Lp}$ or $TrpS_{Lp}$ (pNT562). Protein expression was repressed with 1% glucose. **(B)** Example image of transformation plates from one replicate of the $HipT_{Lp}$ rescue screen with the *E. coli* ASKA genomic library. The ASKA library was pooled and electroporated into BL21-GOLD (DE3) cells containing $HipT_{Lp}$ cloned into the pCDF1-b expression vector. Transformants were plated on solid media containing either IPTG (100 µM) for gene expression or 1% glucose for repression. As a control, the empty vector pCA24N was transformed in an equivalent manner to the pooled library. Library transformations were performed a minimum of three times for each experiment. Individual screening experiments were repeated 3 times. **(C)** Uninduced controls (repressed with 1% glucose) for *L. pneumophila* cells co-expressing $HipT_{Lp}$ (pJB1806) with phosphoproteomic candidates (pNT562) that were either essential, or highly conserved in both *Legionella* and conserved in *E. coli*. **(D)** Uninduced controls (repressed with 1% glucose) for co-expression of $HipT_{Lp}$ with $PheST_{Lp}$ or phosphomimetic mutants of $PheS_{Lp}$(S50A) in *L. pneumophila* cells. Growth curves show the mean ± the standard deviation of 3 biological replicates. Data are representative of 3 independent experiments.

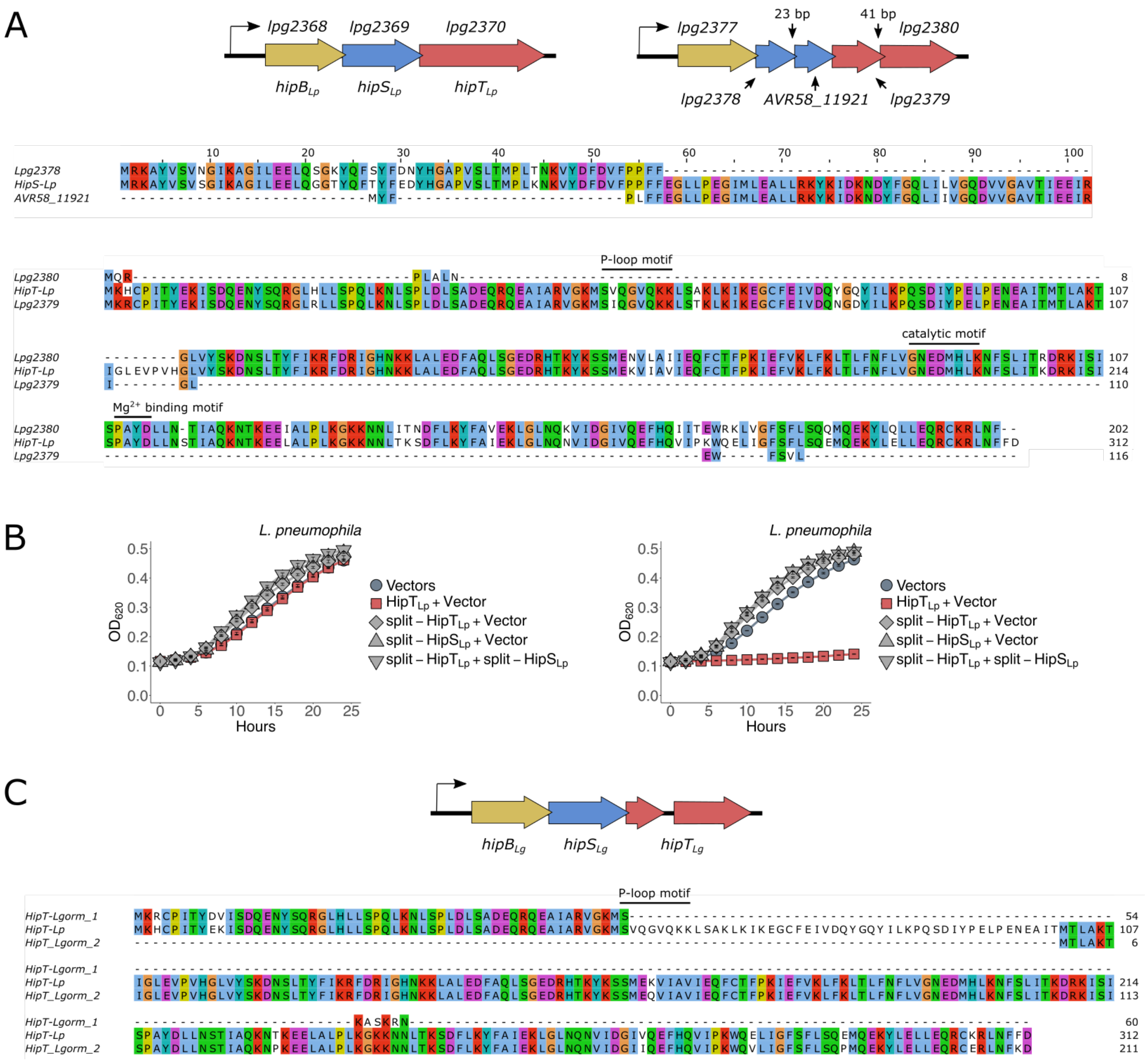

A
lpg2368
lpg2369
lpg2370
hipB_Lp
hipS_Lp
hipT_Lp
23 bp
41 bp
lpg2377
lpg2380
lpg2378
AVR58_11921
lpg2379
Lpg2378
HipS-Lp
AVR58_11921
P-loop motif
Lpg2380
HipT-Lp
Lpg2379
catalytic motif
Mg2+ binding motif
B
L. pneumophila
OD620
Hours
Vectors
HipT_Lp + Vector
split – HipT_Lp + Vector
split – HipS_Lp + Vector
split – HipT_Lp + split – HipS_Lp
C
hipB_Lg
hipS_Lg
hipT_Lg
HipT-Lgorm_1
HipT-Lp
HipT_Lgorm_2

Figure S7. Divergent architectures suggest evolution and decay in *Legionella* HipBST systems. (A) Top: schematic comparing *hipBST*$_{Lp}$ with a homologous pentapartite *hipBST* locus in the *L. pneumophila* genome downstream of the *hipBST*$_{Lp}$ locus. The pentapartite module contains a split-HipS$_{Lp}$ and split-HipT$_{Lp}$ architecture (labelled). Middle: MUSCLE alignment of the split-HipS protein sequences (Lpg2378, AVR58_11921) with HipS$_{Lp}$. Conserved residues are coloured with the Clustal X colour scheme implemented in Jalview. Bottom: MUSCLE alignment of the split-HipT proteins (Lpg2379, Lpg2380) with HipT$_{Lp}$. Conserved motifs are labelled. Conserved residues are coloured with the Clustal X colour scheme implemented in Jalview (> 40%). **(B)** Growth assays of *L. pneumophila* cells expressing split-HipT$_{Lp}$ (pJB1806) and split-HipS$_{Lp}$ (pNT562), either alone or in combination. Uninduced controls (left) were repressed with 1% glucose and cultures were induced with 100 μM IPTG (right). All growth curves show the mean ± the standard deviation of 3 biological replicates. Data are representative of 2 independent experiments. **(C)** Top: schematic of a homologous quadripartite *hipBST* locus in *Legionella gormanii*, which encodes a split-*hipT* fragment that is truncated at the P-loop motif due to a frameshift. A MUSCLE alignment of both split-HipT fragments in *L. gormanii* with HipT$_{Lp}$ is shown. Conserved residues are coloured with the Clustal X colour scheme implemented in Jalview.

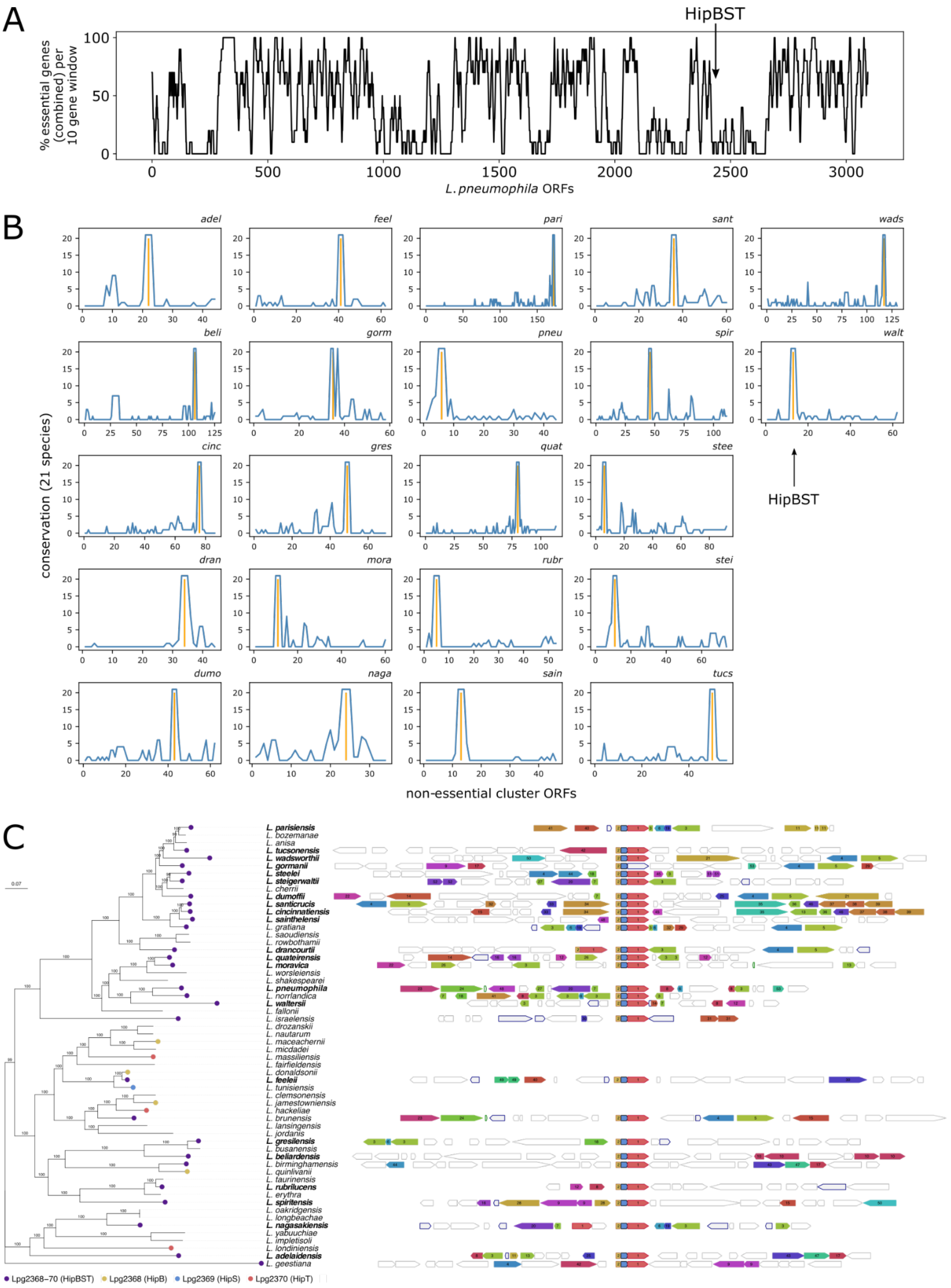

A
HipBST
% essential genes (combined) per 10 gene window
L. pneumophila ORFs
B
adel
feel
pari
sant
wads
beli
gorm
pneu
spir
walt
cinc
gres
quat
stee
dran
mora
rubr
stei
dumo
naga
sain
tucs
HipBST
conservation (21 species)
non-essential cluster ORFs
C
0.07
L. parisiensis
L. bozemanae
L. anisa
L. tucsonensis
L. wadsworthii
L. gormanii
L. steelei
L. steigerwaltii
L. cherrii
L. dumoffii
L. santicrucis
L. cincinnatiensis
L. sainthelensi
L. gratiana
L. saoudiensis
L. rowbothamii
L. drancourtii
L. quateirensis
L. moravica
L. worsleiensis
L. shakespearei
L. pneumophila
L. norrlandica
L. waltersii
L. fallonii
L. israelensis
L. drozanskii
L. nautarum
L. maceachernii
L. micdadei
L. massiliensis
L. fairfieldensis
L. donaldsonii
L. feeleii
L. tunisiensis
L. clemsonensis
L. jamestowniensis
L. hackeliae
L. brunensis
L. lansingensis
L. jordanis
L. gresilensis
L. busanensis
L. beliardensis
L. birminghamensis
L. quinlivanii
L. taurinensis
L. rubrilucens
L. erythra
L. spiritensis
L. oakridgensis
L. longbeachae
L. nagasakiensis
L. yabuuchiae
L. impletisoli
L. londiniensis
L. adelaidensis
L. geestiana
Lpg2368–70 (HipBST)
Lpg2368 (HipB)
Lpg2369 (HipS)
Lpg2370 (HipT)

Figure S8. HipBST$_{Lp}$ is associated with accessory genomic clusters of predicted non-essential genes across *Legionella* species. (A) *L. pneumophila* ORF essentiality inferred from a combination of broth viability (38, 39), conservation across 58 *Legionella* species, and host replication defects in 4 amoebal hosts (39). Data are displayed as 10 gene sliding windows. Troughs indicate predicted accessory genomic regions of non-essential genes. The location of HipBST$_{Lp}$ is indicated. **(B)** Accessory genomic clusters of predicted non-essential genes containing HipBST$_{Lp}$ homologs are shown for 22 *Legionella* species. Four letter abbreviated species names are indicated for each cluster. For every ORF in each cluster (x-axis), conservation in the other 21 *Legionella* species clusters is shown (y-axis). The position of the HipBST homolog in each cluster is indicated with a vertical orange line and labelled for one cluster as an example. **(C)** Graphical display of conserved gene neighbourhoods adjacent to each HipBST system across *Legionella* species as detected by FlaGs. 10 genes upstream and downstream of *hipS* are shown. Genes are numbered based on relative conservation and *Legionella* species with HipBST systems in predicted non-essential clusters are bolded.

REFERENCES

1. Waterhouse AM, Procter JB, Martin DMA, Clamp M, Barton GJ. 2009. Jalview Version 2--a multiple sequence alignment editor and analysis workbench. Bioinformatics 25:1189–1191.

2. Li RW. 2013. Metagenomics and Its Applications in Agriculture, Biomedicine and Environmental Studies. Nova Science Publishers.
